# The tip protein PAAR is required for the function of the type VI secretion system

**DOI:** 10.1128/spectrum.01478-23

**Published:** 2023-10-06

**Authors:** Solène G. Beauvois, Nicolas Flaugnatti, Marianne Ilbert, Marie Boyer, Esther Gavello-Fernandez, Rémi Fronzes, Dukas Jurėnas, Laure Journet

**Affiliations:** 1 Laboratoire d’Ingénierie des Systèmes Macromoléculaires, Institut de Microbiologie, Bioénergies et Biotechnologie, Institut de Microbiologie de la Méditerranée, Aix-Marseille Université - CNRS UMR7255, Marseille, France; 2 Laboratoire de Bioénergétique et Ingénierie des Protéines, Institut de Microbiologie, Bioénergies et Biotechnologie, Institut de Microbiologie de la Méditerranée, Aix-Marseille Université - CNRS UMR7281, Marseille, France; 3 Institut Européen de Chimie et Biologie, University of Bordeaux, Pessac, France; 4 CNRS UMR 5234 Microbiologie Fondamentale et Pathogénicité, Bordeaux, France; Universidad Andres Bello, Santiago, Chile

**Keywords:** type VI secretion system, cryo-electron microscopy, bacterial competition, protein secretion, VgrG, bacterial toxin, zinc

## Abstract

Bacteria are constantly competing to colonize crowded ecological niches, such as the human gut. The type VI secretion system (T6SS) is a critical bacterial weapon in this warfare. It resembles a crossbow with a poisoned arrow allowing bacteria to inject toxic effectors directly into target cells. This machinery is formed by an envelope-spanning complex which recruits the baseplate, an assembly platform allowing the polymerization of a contractile structure. The tail consists of a tube surrounded by a sheath and topped by the needle complex composed of the VgrG and PAAR proteins. In the enteric pathogen enteroaggregative *Escherichia coli* (EAEC), the Tle1 phospholipase toxin ensures the antibacterial activity of the T6SS-1. For its transport, Tle1 interacts directly with the trimeric spike protein VgrG. However, the importance and the function of the tip protein PAAR in the T6SS remain unclear. Here, we characterized the PAAR protein of EAEC using biochemical, fluorescence microscopy, and antibacterial competition approaches. Using pull-down assays and cryo-electron microscopy analysis of the (VgrG)_3_-(Tle1)_3_-PAAR complex, we show that PAAR tops and closes the β-prism structure of the VgrG spike. The PAAR protein structure is further tightened by the zinc atom coordinated via conserved residues essential for its function. We provide evidence that PAAR is necessary for T6SS-1-mediated killing due to its requirement for proper T6SS baseplate assembly and further sheath polymerization. Our results suggest that the PAAR protein is an essential component of T6SS.

**IMPORTANCE:**

The type VI secretion system (T6SS) is a bacterial contractile injection system involved in bacterial competition by the delivery of antibacterial toxins. The T6SS consists of an envelope-spanning complex that recruits the baseplate, allowing the polymerization of a contractile tail structure. The tail is a tube wrapped by a sheath and topped by the tip of the system, the VgrG spike/PAAR complex. Effectors loaded onto the puncturing tip or into the tube are propelled in the target cells upon sheath contraction. The PAAR protein tips and sharpens the VgrG spike. However, the importance and the function of this protein remain unclear. Here, we provide evidence for association of PAAR at the tip of the VgrG spike. We also found that the PAAR protein is a T6SS critical component required for baseplate and sheath assembly.

## INTRODUCTION

Bacteria constantly compete for colonization of the ecological niches granting the access to nutrient sources. The best armed bacteria will have greater chances to win this warfare and plenty of weapons are deployed to succeed in this competition. The type VI secretion system (T6SS) is one of the crucial weapons allowing bacteria to inject toxic effectors directly into the target cells. There are two categories of T6SS toxins. T6SS periplasmic-acting toxins can degrade peptidoglycan and membrane phospholipids or form pores in the inner membrane. T6SS cytoplasmic effectors target DNA, deplete energy resources, inhibit cell division, or inhibit translation (1 – 4). T6SS can also target eukaryotic cells, employing effectors mainly targeting the eukaryotic cytoskeleton and membranes (1, 2, 5).

The T6SS machinery is composed of 13–14 core components arranged in gene clusters (6, 7). The architecture can be subdivided into three subcomplexes: the membrane complex, the baseplate, and the tail tube/sheath complex (TTC). The membrane complex is a 1.7 MDa complex that anchors the system to the bacterial envelope (8, 9). It is composed of outer membrane lipoprotein TssJ and inner membrane proteins TssM and TssL (in a 15:10:10 stoichiometry). The membrane complex recruits the baseplate which serves as an assembly platform required for the polymerization of the contractile phage tail-like tubular structure (10 – 15). The baseplate is assembled from six wedges composed of Tss-K, -E, -F, -G proteins that arrange around a central hub, formed by the trimeric spike protein VgrG (14, 15). With the help of TssA protein, the baseplate directs the polymerization of the contractile TTC structure (11, 16). The TTC is composed of an internal tube made of Hcp protein hexamers, surrounded by a contractile sheath, made of TssB and TssC subunits. This cytoplasmic tail is about 1 µm long and assembles within roughly 40 s through the TssA protein-coordinated polymerization of Hcp and TssB/C components (10, 16, 17). Upon contact with prey, the sheath contracts and expels the tube surmounted by the spike, together with the effectors into the target cell. Indeed, the effectors are fused or interact with these structural components (1, 3, 4, 18, 19). The enteroaggregative *Escherichia coli* T6SS cluster 1 (EAEC T6SS-1) delivers the phospholipase toxin Tle1 (20). Three Tle1 cargo effectors are loaded on the sides of the VgrG trimer via direct protein-protein interactions (20, 21). The N-terminal β-strand of Tle1 interacts with the C-terminal transthyretin (TTR) extension of VgrG through fold complementation. VgrG-Tle1 interaction is further stabilized by additional zones of contact on the sides and at the base of the gp5-like domain of VgrG. The Tle1 phospholipase A1 activity is inhibited by the interaction with the VgrG protein (21). Almost the entire T6SS, except the membrane complex, structurally resembles contractile bacteriophages, such as T4 (22 – 25). The T6SS spike protein VgrG is structurally homologous to the T4 puncturing device composed of gp27 and gp5 protein trimers (22, 23). Precisely, the triangular base of VgrG also known as a hub domain is homologous to gp27. It is followed by an oligonucleotide/oligosaccharide-binding (OB)-fold domain which is homologous to gp5* domain, and extended by a β-helical gp5C-like domain (21 – 23, 26, 27). The blunt end of the VgrG β-helical prism is covered by a single PAAR protein that knots the trimeric structure into a uniform sharp tip (28). PAAR, named after proline-alanine-alanine-arginine repeats, is structurally homologous to the gp5.4 protein of bacteriophage T4 that similarly caps the central spike complex formed by gp5C (12, 28, 29).

PAAR was shown to be either important or absolutely required for T6SS secretion or killing activity in different systems (28, 30 – 32). Its role remains to be determined, although some hypotheses proposed that it stabilizes the trimeric VgrG complex or sharpens the T6SS for target cell puncturing.

Here, we report structural and functional analysis of EC042_4537 gene of the EAEC T6SS-1 that is predicted to code for a PAAR protein. Single particle cryo-electron microscopy (cryo-EM) analysis of the VgrG-Tle1-PAAR complex from EAEC revealed that PAAR caps the blunt end of the VgrG needle. Inductively coupled plasma optical emission spectrometry (ICP-OES) analysis of purified PAAR protein shows that a zinc atom is coordinated by conserved histidines and cysteines (H14, H46, C41, C74). Bacterial competition assays indicate that PAAR protein and its metal coordinating residues H46, C41, and C74 are essential for T6SS-1 antibacterial activity. Finally, using fluorescence microscopy, we further demonstrate that PAAR is required for correct baseplate localization and thus for sheath assembly. Taken together, our results confirm that the PAAR protein is an essential core component of the T6SS.

## RESULTS

### PAAR caps the C-terminal needle domain of VgrG spike complex

**Fig 1:**
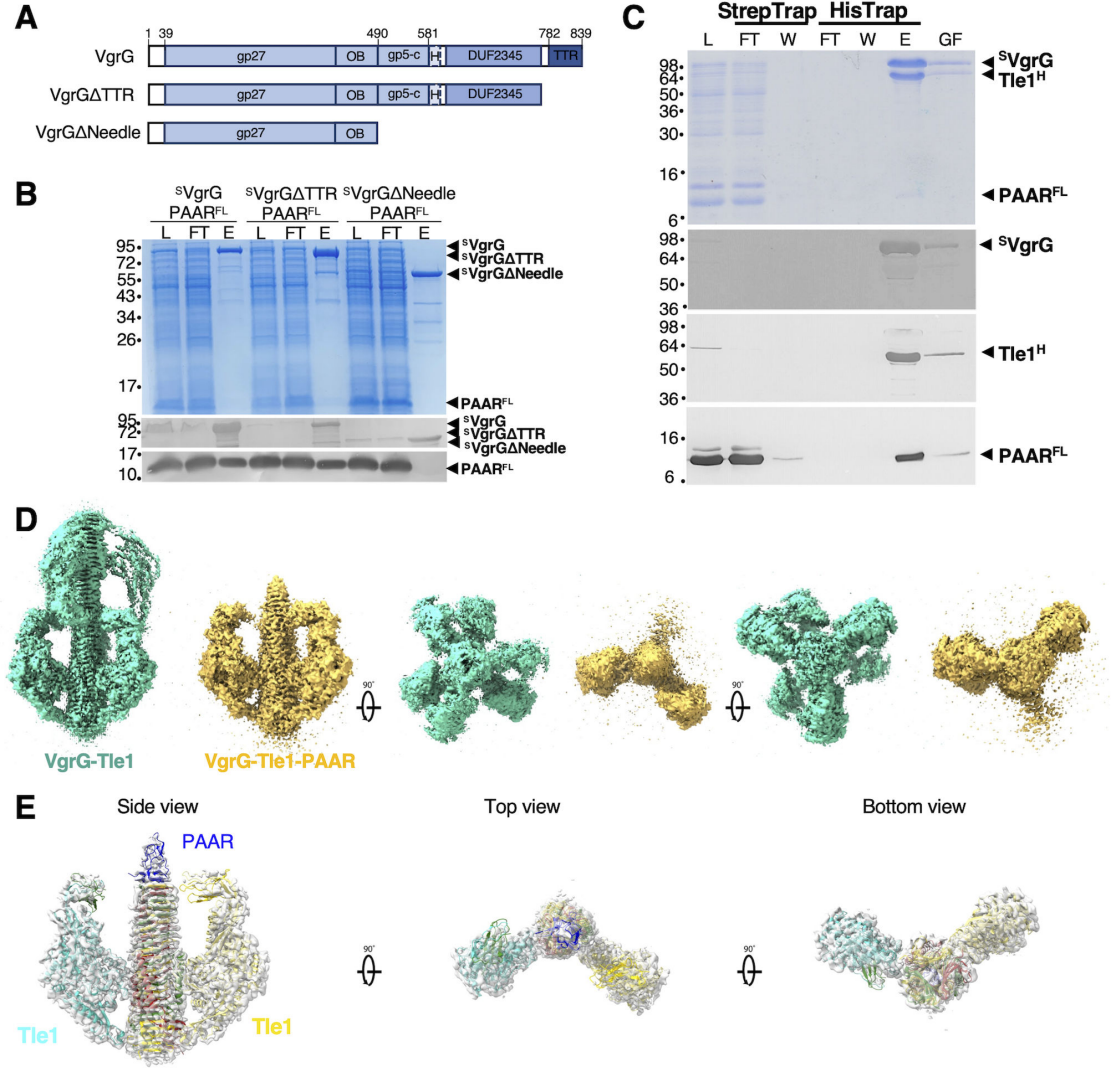
PAAR interacts with the VgrG needle domain and caps the VgrG-Tle1 complex. (**A**) Schematic representation of the EAEC VgrG1 protein and the truncated variants used for pull-down assays with PAAR. The different domains and their boundaries are indicated—base domain gp27; OB fold domain; β-helical domain gp5-C; H, helix; DUF2345 domain; TTR, transthyretin-like domain. (**B**) Pull-down assays. Lysates of BL21(DE3) cells co-producing FLAG-tagged PAAR (PAAR^FL^) and Strep-tag II-tagged VgrG (^S^VgrG) or truncated versions of VgrG (^S^VgrG ΔTTR, ^S^VgrG ΔNeedle) were loaded (**L**) on a Strep-Tactin column. After washing steps, desthiobiotin-eluted (**E**) and flow through (FT) fractions were analyzed by SDS-PAGE and Coomassie blue staining (upper panel), immunoblot using anti-StrepII (middle panel) and anti-FLAG (lower panel). (**C**) VgrG-PAAR-Tle1 complex purification. SDS-PAGE analysis and Coomassie blue staining (top panel), immunoblot using anti-StrepII (upper middle panel), anti-His (lower middle panel), and anti-FLAG (bottom panel) of the purification steps. Cell lysate (**L**) co-producing Strep-tag II-VgrG (^S^VgrG), Tle1-6×His (Tle1^H^), and PAAR-FLAG (PAAR^FL^) was loaded onto a StrepTrapHP column. After washing (**W**), the material was eluted with desthiobiotin directly into a HisTrap column. The imidazole-eluted material (**E**) was then loaded onto a Superose 6 10/300 gel filtration column (GF). Ten microliters of each fraction was loaded onto the gel. The molecular weight markers (in kDa) are indicated on the left and the positions of the different proteins are indicated on the right. (**D**) Cryo-EM density maps of VgrG-Tle1 and VgrG-Tle1-PAAR complex. Left: Cryo-EM density maps of VgrG needle bound to Tle1 (in green) corresponding to a (VgrG)_3_-(Tle3)_3_ tip-to-tip dimer complex. Right: VgrG needle bound to Tle1 in the presence of the PAAR protein (in yellow). In the presence of PAAR, most particles correspond to VgrG needle bound to one or two Tle1 (represented here) and no VgrG needle tip-to-tip dimerization. 2D classes are presented in Fig. S1. (**E**) VgrG-Tle1-PAAR complex model. AlphaFold2 co-folding model of the needle domain of VgrG (three copies, green, yellow, red) with PAAR (blue) was aligned with the VgrG-Tle1 model (PDB:6SJL) merging on the needle domain. This model was fitted in the cryo-EM density maps (4.2 Å, two Tle1 visible).

T6SS-1 encodes a unique predicted PAAR protein, downstream the *tli1 g*ene coding for immunity protein against the Tle1 toxin (20). Our previous cryo-EM structure of the EAEC (VgrG)_3_-(Tle1)_3_ complex revealed that the toxins are loaded on the sides of the β-prism of the VgrG spike, leaving the top of the β-prism free to accommodate the PAAR protein (21). To test this hypothesis, we aimed to determine where precisely does the PAAR localize on this structure. We heterologously co-produced PAAR^FLAG^ (PAAR^FL^) and ^Strep^VgrG (^S^VgrG), as well as its truncated versions deleted for the TTR domain (^S^VgrG1-778 called ^S^VgrGΔTTR) or the whole β-prism (needle) part (^S^VgrG1-490 called ^S^VgrGΔNeedle) and tested direct interactions by streptactin pull-down assays (Fig. 1A and B). As shown in Fig. 1B, PAAR^FLAG^ co-precipitated with ^S^VgrG and ^S^VgrGΔTTR but not with VgrG deleted for the whole β-prism (^S^VgrGΔNeedle). We conclude that PAAR directly interacts with the C-terminal domain of VgrG without implication of the TTR domain. A complete spike complex loaded with toxin PAAR^FL^-^S^VgrG-Tle1^H^ was purified using a double-affinity and size exclusion chromatography (Fig. 1C) and analyzed using single particle cryo-EM (Fig. 1D and E; Fig. S1A). For comparison, we have repeated the analysis of a previously reported ^S^VgrG-Tle1^H^ complex without PAAR purified and analyzed in the same conditions. As previously reported, the (VgrG)_3_-(Tle1)_3_ complexes dimerized through interactions at the top of the VgrG needle to produce violin body-shaped 2D classes [(21), Fig. 1D; Fig. S1B]. Such dimerization was completely absent in the VgrG-Tle1-PAAR complex, suggesting that PAAR prevents the two blunt ends of VgrG β-prisms from sticking together (Fig. 1D; Fig. S1A). A single conical density on top of the trimeric VgrG needle prism was readily recognizable and likely corresponds to the PAAR protein (Fig. 1D). Surprisingly, the 2D classification revealed heterogeneity in particles yielding classes where three, two, one, or none of the Tle1 toxins were visible judging from the side and the top views (Fig. S1A). This is in contrast with the violin body-shaped 2D classes of the (VgrG)_3_-(Tle1)_3_ complex (without PAAR) where sharp densities for all the toxins were typically observed (Fig. S1B) (21). This suggests that the toxin positions are locked by the unnatural dimerization of the (VgrG)_3_-(Tle1)_3_ complexes that occurs due to the absence of the PAAR protein. This dimerization seems to lock the TTR regions in fixed position that are extended from the VgrG structure and hang on the flexible linkers not visible in the structure (21). It is therefore likely that the toxins loaded on the VgrG are indeed quite flexible, which explains the partial absence of the Tle1 densities in VgrG-Tle1-PAAR complex where TTRs are not fixed.

Due to very small size and lack of symmetry, the resolution was not sufficient to build an experimental atomic model of the PAAR protein in the obtained cryo-EM density maps. We have therefore produced an AlphaFold2 co-folded model of the needle domain of VgrG together with PAAR and aligned it to the experimental VgrG-Tle1 model (PDB:6SJL). The resulting complete model could be readily docked into best resolved cryo-EM 3D volumes representing (VgrG)_3_-(Tle1)_2_-PAAR (4.2 Å) or (VgrG)_3_-(Tle1)_1_-PAAR (3,7 Å) structures (Fig. 1E; Fig. S1C). The triangular shape of the PAAR model perfectly fits into the extra conical density that we assigned to PAAR (Fig. 1E).

### PAAR binds a zinc atom via conserved cysteine and histidine residues

**Fig 2:**
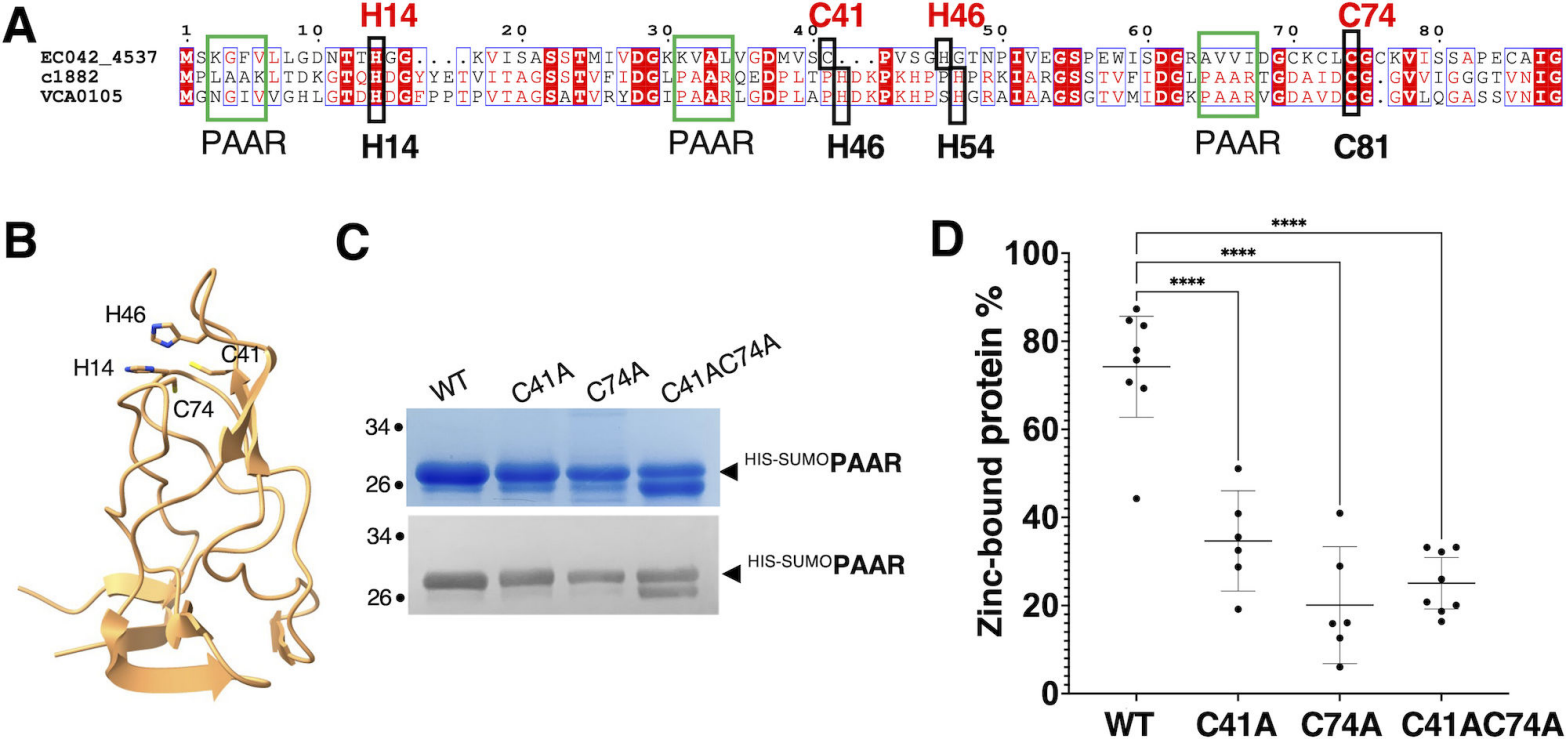
PAAR binds a zinc atom involving conserved His and Cys residues. (**A**) Alignment of amino acid sequences of PAAR^EAEC^ (EC042_4537), PAAR from *E. coli* CFT073 (c1882), and *Vibrio cholerae* (VCA0105). PAAR motifs are shown in green boxes, His and Cys residues involved in zinc binding characterized in reference (28) are indicated in black, below the alignment. H14, C41, H46, and C74, the predicted residues involved in metal binding in PAAR^EAEC^ are indicated in red above the alignment. (**B**) AlphaFold2 model of PAAR highlighting His14, Cys41, His46, and Cys74 predicted metal-binding residues. PAAR was co-modeled with VgrG needle part and only PAAR is shown here. (**C**) SDS-PAGE followed by Coomassie blue staining (upper panel) and immunoblot using anti-His-Tag (lower panel) of purified wild-type ^His-SUMO^-PAAR proteins and mutated versions (^His-SUMO^-PAAR^C41A^, ^His-SUMO^-PAAR^C74A^, and ^His-SUMO^-PAAR^C41AC74A^). The molecular weight markers (in kDa) are indicated on the left. (**D**) Zinc content analysis using ICP-OES of purified ^His-SUMO^-PAAR (WT) and cysteine mutants (^His-SUMO^-PAAR^C41A^, C41A; ^His-SUMO^-PAAR^C74A^, C74A; ^His-SUMO^-PAAR^C41AC74A^, C41AC74A). Statistical significance was calculated using ordinary one-way analysis of variance (ANOVA) followed by Dunnett’s multiple comparisons test using GraphPad Prism (GraphPad Software). ****P < 0.0001. Each value represents the mean of three technical replicates of two to four different fractions of two (C41A, C74A) to three different purification preparations (WT, C41AC74A).

The 88 amino acid length protein PAAR (EC042_4537) from EAEC T6SS-1 belongs to class 1 PAAR family (28) carrying a PAAR_CT_2 domain (cd14744 of National Center for Biotechnology Information [NCBI] Conserved Domain Database). The PAAR^EAEC^ amino acid sequence was aligned with the sequences of structurally characterized PAAR proteins from *Vibrio cholerae* and *E. coli* CFT073 (Fig. 2A) (28). These two proteins were shown to bind a zinc atom that is coordinated by three histidines (His) and one cysteine (Cys) localized at the extremity of the tip (28). Three motifs with hydrophobic residues that could functionally resemble PAAR motifs (Pro-Ala-Ala-Arg) could be identified using sequence alignment (Fig. 2A). In the PAAR^EAEC^ protein, two His (His14 and His46) and two Cys (Cys41 and Cys74) residues are conserved and localize at the sharp end of the AlphaFold2 structural model of PAAR (Fig. 2B). We therefore hypothesized that these residues could be involved in metal binding (Fig. 2A and B) as previously reported for *V. cholerae* and *E. coli* PAAR proteins (28). PAAR^EAEC^ protein was fused to a SUMO protein to increase its solubility and a 6×histidine tag for purification with affinity chromatography (Fig. 2C). ICP-OES analysis of the purified protein showed that 74.2% (±13.7 SD) of PAAR^EAEC^ contains zinc (Fig. 2D). Only small amounts (<0.75% of total protein) of iron, copper, or nickel were found in the samples (Fig. S2). PAAR^EAEC^ proteins with single and double alanine substitutions in conserved cysteines C41 and C74 exhibited a significant decrease in zinc content compared to the wild type (WT) (Fig. 2D). Only 34.7% (±10.9 SD) of PAAR^C41A^, 20.1% (±12. 7 SD) of PAAR^C74A^, and 25.1% (±7.0 SD) of PAAR^C41AC74A^ were found to coordinate a zinc atom (Fig. 2D). To rule out the possibility that the overall fold of the small proteins was impaired, we have assayed the interaction of the mutants with VgrG. The PAAR^EAEC^ proteins with single C41A and C74A, and to lesser extent double C41AC74A, substitutions retained their capacity to interact with VgrG, suggesting that these mutations mainly affect metal coordination (Fig. S3). Overall, these results indicate that PAAR^EAEC^ protein binds a zinc atom via at least the two conserved cysteines C41 and C74.

### PAAR is required for the antibacterial activity of T6SS

**Fig 3:**
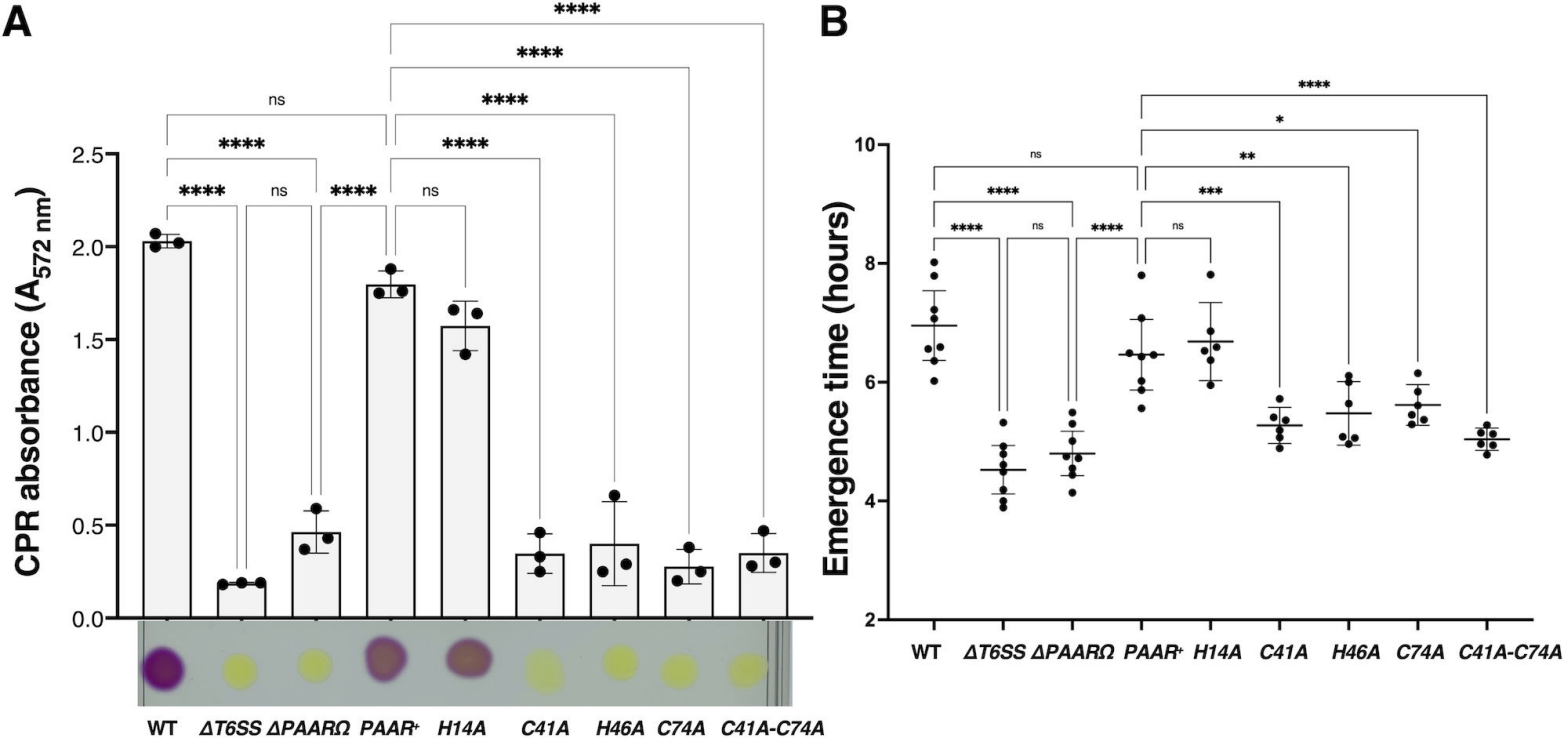
PAAR is necessary for T6SS-1-mediated killing. (**A**) Antibacterial competition assay by the colorimetric method. The T6SS-1 function of the WT, ΔPAARΩ, and ΔPAARΩ complemented with PAAR (PAAR^+^) or PAAR Cys and His mutant strains (using pBAD_33_-PAAR^VSVG^ and corresponding PAAR Cys and His mutant plasmids derivatives) was tested by assessing their ability to kill W3110 *E. coli* K12 bacterial prey. Killing efficiency was monitored by observing degradation of yellow CPRG into purple CPR by free β-galactosidase released from lysed prey cells after being exposed to the predator cells. CPR absorbance (*A*_572nm_, upper graph) was measured from the spots (lower panel). The means of three biological replicates are indicated. The error bars represent standard deviation. Statistical significance was calculated using ordinary one-way ANOVA followed by Dunnett’s multiple comparisons test using GraphPad Prism (GraphPad Software). ****P < 0.0001. (**B**) Antibacterial competition assay by the emergence time method. The T6SS-1 function of the WT, ΔPAARΩ, and ΔPAARΩ complemented with PAAR or PAAR-Cys and His-mutant strains was tested by assessing their ability to kill W3110 *E. coli* K12 bacterial prey. Killing efficiency was quantified by measuring the time (in hours) needed for prey cells exposed to predator cells to regrow in selective media to *A*_600_ = 0.4. Statistical significance was calculated using ordinary one-way ANOVA followed by Dunnett’s multiple comparisons test using GraphPad Prism. ****P < 0.0001; ***P = 0.0009; **P = 0.0092; *P = 0.0401, ns = 0.9711 (ΔT6SS vs ΔPAARΩ), ns = 0.9967 (PAAR^+^ vs H14A).

In the systems with multiple T6SS tip complexes, at least one PAAR protein was shown to be required for functionality of the T6SS but some reports suggested that it is not always necessary (28, 30 – 33). Our model provides unambiguous case to study the role of PAAR, since only one tip complex exists in EAEC T6SS-1 and the loading of toxins on the VgrG spike does not require PAAR (Fig. 1D). To determine the importance of PAAR protein in the function of T6SS of EAEC, we have tested the capacity of a *PAAR* deletion mutant (ΔPAARΩ) to kill *E. coli* W3110 prey, using two different methods (34) (Fig. 3). First, the predator strains and the prey cells were grown separately in T6SS-1 inducing medium (SIM) before being mixed at a 1:4 ratio (for colorimetric method) or 4:1 ratio (for emergence time method) and spotted on SIM agar plates. In the case of the colorimetric method, after 2 h of contact, yellow chlorophenol-red β-D-galactopyranoside (CPRG) substrate was deposited on the mixed-cell spots. CPRG substrate was degraded into purple chlorophenol red (CPR) product by β-galactosidase released from the prey cells upon cell lysis induced by the secreted phospholipase toxin Tle1 by the wild-type strain. On the contrary, competition with deletion strain of the whole T6SS-1 cluster (ΔT6SS) used for control retains yellow color of the mixed spot reporting the absence of cell lysis. Interestingly, the ΔPAARΩ mutant was not able to kill the W3110 strain. However, its antibacterial activity could be restored by complementing the expression of PAAR protein from a plasmid (PAAR^+^) (Fig. 3A). To perform quantitative analysis, we have further pursued the emergence time method (34). After 2 h of contact, each cell spot was scraped from agar plates and diluted into a liquid medium selective for prey cells. The re-growth of prey cells was evaluated by measuring *A*_600_ for 15 h using a microplate reader. The emergence time, being the time for the cultures to reach an *A*_600_ equal to 0.4, was plotted for each condition (Fig. 3B). After being exposed to an EAEC WT strain or a complemented ΔPAARΩ mutant strain, prey cells took 7 h to reach an *A*_600_ of 0.4, indicating very little prey cells have survived to restore the growth in culture. On the contrary, prey cells exposed to ΔT6SS-1 or ΔPAARΩ mutants took less than 5 h to reach the same *A*_600_, indicating that much more prey cells remained viable in the mixed-cell spot. Both methods confirm that the PAAR protein is required for the EAEC T6SS-1 antibacterial activity. We have also tested whether expression of PAAR with the single amino acid substitutions in conserved metal coordination residues could complement lack of wild-type PAAR protein. Interestingly, except for histidine H14, substitutions in all the other residues predicted to be involved in zinc binding, i.e., C41, C74, and H46 could not complement PAAR deletion. This further suggests the importance of zinc binding at the tip of the PAAR for its function (Fig. 3). The functionality of H14 mutant may suggest that it is not involved in zinc binding. However, it is not unusual that the deletion of a single metal coordinating residue is not enough to affect metal binding. The Cys76 is well located in the predicted structure to compensate the absence of His14 for metal binding (Fig S4A). We thus constructed and analyzed C76A and His14AC76A mutants. Both H14A and C76A single mutants, but not the double mutant H14AC76A, could complement PAAR deletion in the killing assay (Fig S4B). Accordingly, the zinc content of the double mutant H14AC76A, but not that of C76A or H14A, seems to be impaired compared to the wild type (Fig S4C). Altogether, these data suggest that C41, C74, H46, and likely H14 (or C76) are important for zinc binding and function of PAAR^EAEC^. As a control, we have assayed alanine substitutions of other cysteine residues of PAAR^EAEC^ protein, i.e., C70 and C72, that are positioned elsewhere in the structure and are thus not likely to contribute to metal binding (Fig. S4AB). As expected, no effect on bacterial killing was observed using those mutants. The production in ΔPAARΩ of the different versions of PAAR protein from pBAD plasmids was confirmed by Western blot (Fig. S4F).

### PAAR is required for the polymerization of T6SS sheath

**Fig 4:**
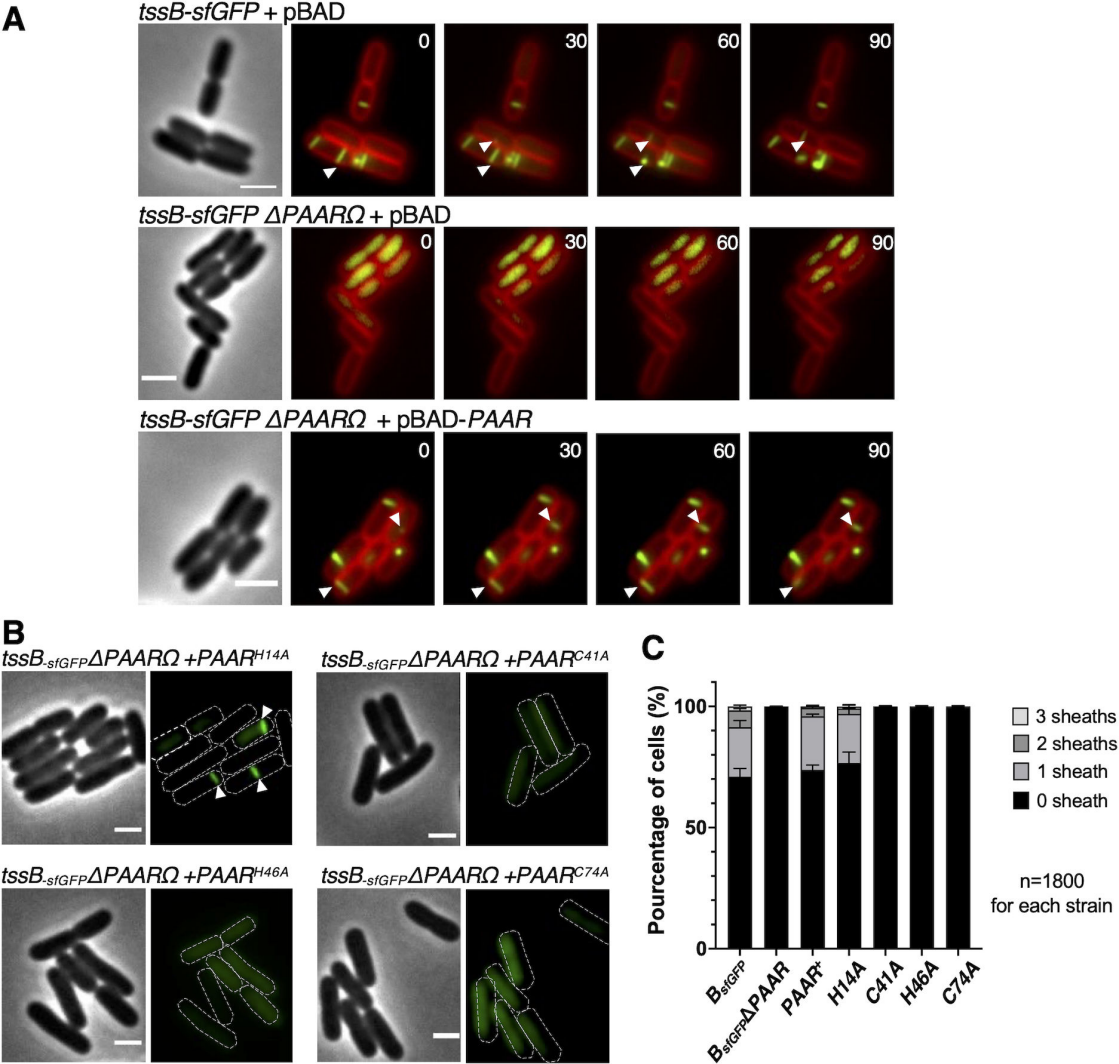
PAAR is necessary for T6SS assembly. (**A**) Fluorescence microscopy of EAEC *tssB-sfGFP* strains transformed with the empty vector pBAD_33_ (*tssB-sfGFP* + pBAD), EAEC *tssB-sfGFP ΔPAARΩKan* transformed with the empty vector pBAD_33_ (*tssB-sfGFP ΔPAARΩ* + pBAD), and EAEC *tssB-sfGFP ΔPAARΩ* complemented in *trans* with pBAD_33_-PAAR^VSVG^ plasmid (*tssB-sfGFP ΔPAARΩ* + pBAD-*PAAR*). Phase contrast images are shown on the left (scale bar = 2 µm). Fluorescence images of TssB-sfGFP (green) and membrane dye FM4-64 (red) are shown on the right. Pictures taken every 30 s are shown from left to right. White arrows indicate dynamic sheath assembly and/or contraction events. (**B**) Fluorescence microscopy of EAEC *tssB-sfGFP ΔPAARΩKan* strain transformed with pBAD_33_-PAAR^H14A-VSVG^, pBAD_33_-PAAR^C41A-VSVG^, pBAD_33_-PAAR^H46A-VSVG^, or pBAD_33_-PAAR^C74A-VSVG^. Phase contrast images are shown on the left (scale bar = 2 µm). Fluorescence images of TssB-sfGFP (green) are shown on the right. Cells are outlined in white, white arrows indicate dynamic sheath assembly and/or contraction events. (**C**) Quantification of the number of extended sheaths per cell from the strains *tssB-sfGFP* + pBAD (B_sfGFP_), *tssB-sfGFP ΔPAARΩ* + pBAD (B_sfGFP_Δ*PAAR), tssB-sfGFP ΔPAARΩ* + pBAD-PAAR^VSVG^ (PAAR^+^), and *tssB-sfGFP ΔPAARΩ* transformed with pBAD_33_-PAAR^H14A-VSVG^ (H14A), pBAD_33_-PAAR^C41A-VSVG^ (C41A), pBAD_33_-PAAR^H46A-VSVG^ (H46A), or pBAD_33_-PAAR^C74A-VSVG^ (C74A) analyzed in A and B. The total number of analyzed cells (n) from three independent biological replicates is indicated. The error bars represent standard deviation.

We wanted to further understand whether the killing defect in the absence of PAAR is due to its requirement for T6SS assembly or in the later stages such as target cell penetration. We have therefore used fluorescence microscopy to follow the dynamics of TssB sheath protein fused to the superfolder green fluorescent protein (sfGFP) that allows observation of sheath elongation and contraction events. Time lapse fluorescence microscopy revealed that as compared to wild-type strain that could assemble T6SS sheaths, the PAAR mutant (ΔPAARΩ) displayed a diffuse fluorescence indicating that TssB-sfGFP proteins are produced but T6SS sheaths are not assembled (Fig. 4A). However, the complementation of PAAR protein expression from a plasmid (ΔPAARΩ + pBAD–*PAAR*) could restore the wild-type phenotype (Fig. 4A) which confirms that PAAR protein is necessary for the assembly of T6SS sheaths in EAEC. In addition, PAAR function is not affected by the presence of a C-terminal vesicular stomatitis virus (VSV-G) tag (Fig. 3 and Fig. 4A).

To assess the role of conserved cysteines and histidines of PAAR^EAEC^, that we have shown to be involved in the binding of a zinc atom (C41/C74/H46) and required for T6SS-mediated killing (C41/C74/H46), we tried to complement the *tssB-sfGFP ΔPAARΩ* by plasmid expression of PAAR^H14A^, PAAR^C41A^, PAAR^H46A^ or PAAR^C74A^, as well as PAAR^C76A^ or PAAR^H14AC76A^. Strains producing PAAR^C41A^, PAAR^H46A^, or PAAR^C74A^ showed diffuse fluorescence confirming that these three residues are crucial for PAAR function in the T6SS sheath assembly (Fig. 4BC). In contrast, wild type-like sheath assembly was observed by complementing with PAAR^H14A^, PAAR^C70A^, PAAR^C72A^, or PAAR^C76A^ which is consistent with antibacterial competition assays indicating that these residues do not play an important role in the function of PAAR protein (Fig. 4BC; Fig. S4E). Again, expression of the PAAR^H14AC76A^ double mutant could not restore sheath assembly, suggesting that the presence of at least H14 or C76 residue in PAAR is necessary for T6SS function.

### PAAR is necessary for the correct localization of the T6SS baseplate, but not of the membrane complex

**Fig 5:**
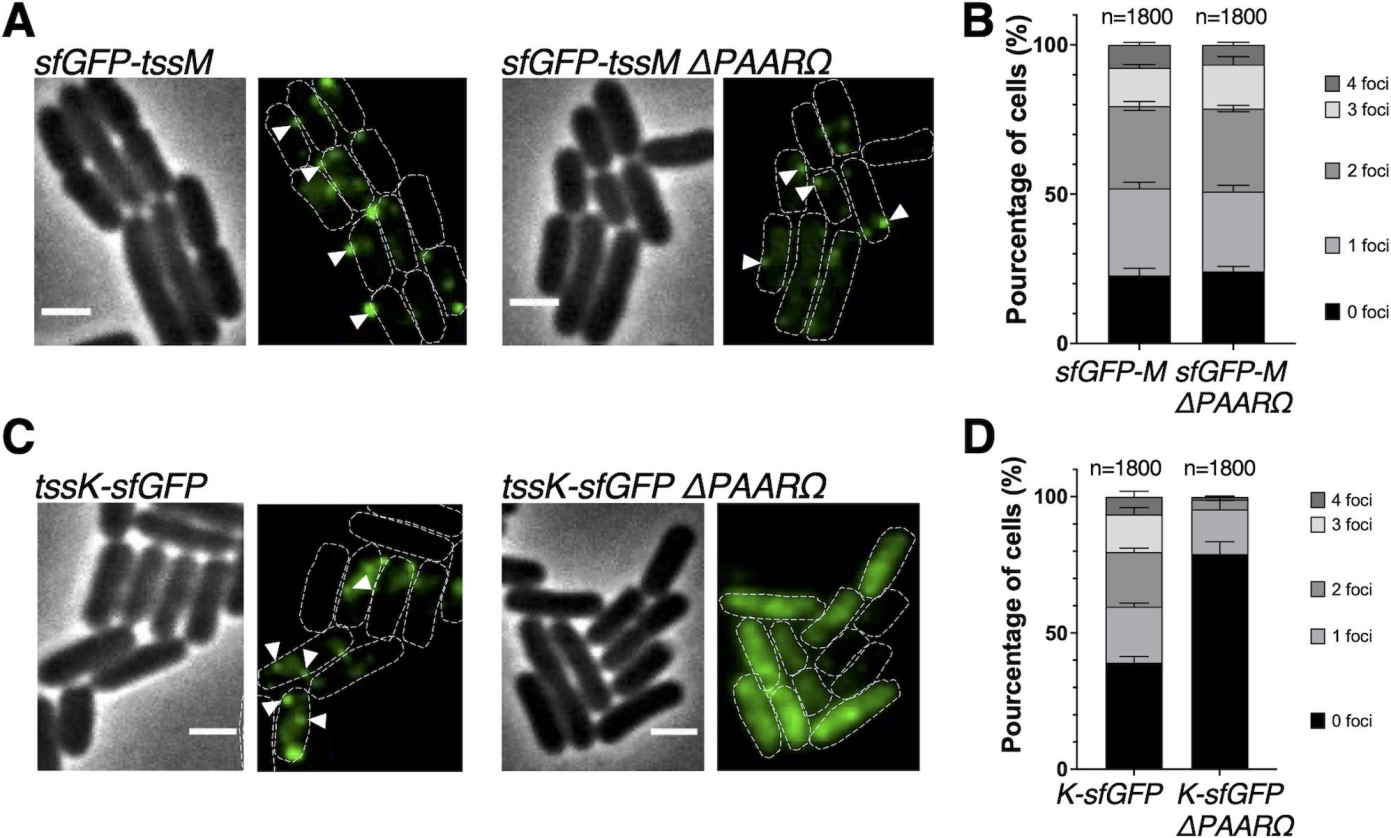
PAAR is necessary for TssK but not TssM localization. (**A**) Fluorescence microscopy analysis of *sfGFP-tssM and sfGFP-tssM ΔPAARΩKan*. Phase contrast images are shown on the left (scale bar = 2 µm) and fluorescence images of sfGFP (green) are shown on the right. Cells are outlined in white, white arrows indicate fluorescent foci localized at the membrane. (**B**) Quantification of the number of foci per cell from *sfGFP-tssM* and *sfGFP-tssM ΔPAARΩKan*. The total number of analyzed cells (n) from three independent biological replicates is indicated. The error bars represent standard deviation. (**C**) Fluorescence microscopy analysis of *tssK-sfGFP and tssK-sfGFP ΔPAARΩKan*. Phase contrast images are shown on the left (scale bar = 2 µm) and fluorescence images of sfGFP (green) are shown on the right. Cells are outlined in white, white arrows indicate fluorescent foci localized at the membrane. (**D**) Quantification of the number of foci per cell from *tssK-sfGFP and tssK-sfGFP ΔPAARΩKan*. The total number of analyzed cells (n) from three independent biological replicates is indicated. The error bars represent standard deviation.

The membrane complex recruits the baseplate complex that is required for the coordinated assembly of the tube and sheath components (8, 11, 13, 16). To determine at which step of the T6SS biogenesis PAAR protein is required, we followed the localization of the membrane complex component TssM and the baseplate component TssK by fluorescence microscopy. We observed the wild-type and *PAAR* mutant (ΔPAARΩKan) cells producing sfGFP-TssM or TssK-sfGFP (Fig. 5). As described previously, in T6SS-active strains, the sfGFP-TssM and TssK-sfGFP present foci at the membrane [(8, 11), Fig. 5]. We noted that in the absence of PAAR, TssM was correctly localized at the membrane, suggesting that PAAR is not required for proper localization of sfGFP-TssM and thus for membrane complex formation (Fig. 5AB). However, the localization of TssK-sfGFP became diffuse in the absence of PAAR, suggesting that PAAR is required for proper formation of the baseplate and its recruitment to the membrane complex (Fig. 5CD). Consequently, PAAR was also required for downstream events—the TssB/C sheath assembly (Fig. 4A) and the T6SS-1-mediated killing (Fig. 3).

## DISCUSSION

There is a long-standing debate about the necessity of the PAAR protein for the T6SS-mediated secretion and killing. Reports using different bacteria as T6SS models yielded contradictory results (28, 30 – 33). Most recently, it was concluded that PAAR is only necessary when transporting toxic effectors (32). In this work, we have employed a model system that can tackle this question since (i) under well-described conditions, (ii) it has only one T6SS firing complex (iii) which has a single PAAR protein that is (iv) uncoupled from toxin loading. Using the well-studied EAEC model, we have demonstrated that PAAR^EAEC^ protein is required for antibacterial activity mediated by the T6SS-1. Our results indicate that PAAR^EAEC^ is indispensable for T6SS-mediated killing by participating in the upstream event—the recruitment of the baseplate that allows polymerization of contractile sheath of T6SS (Fig. 5).

**Fig 6:**
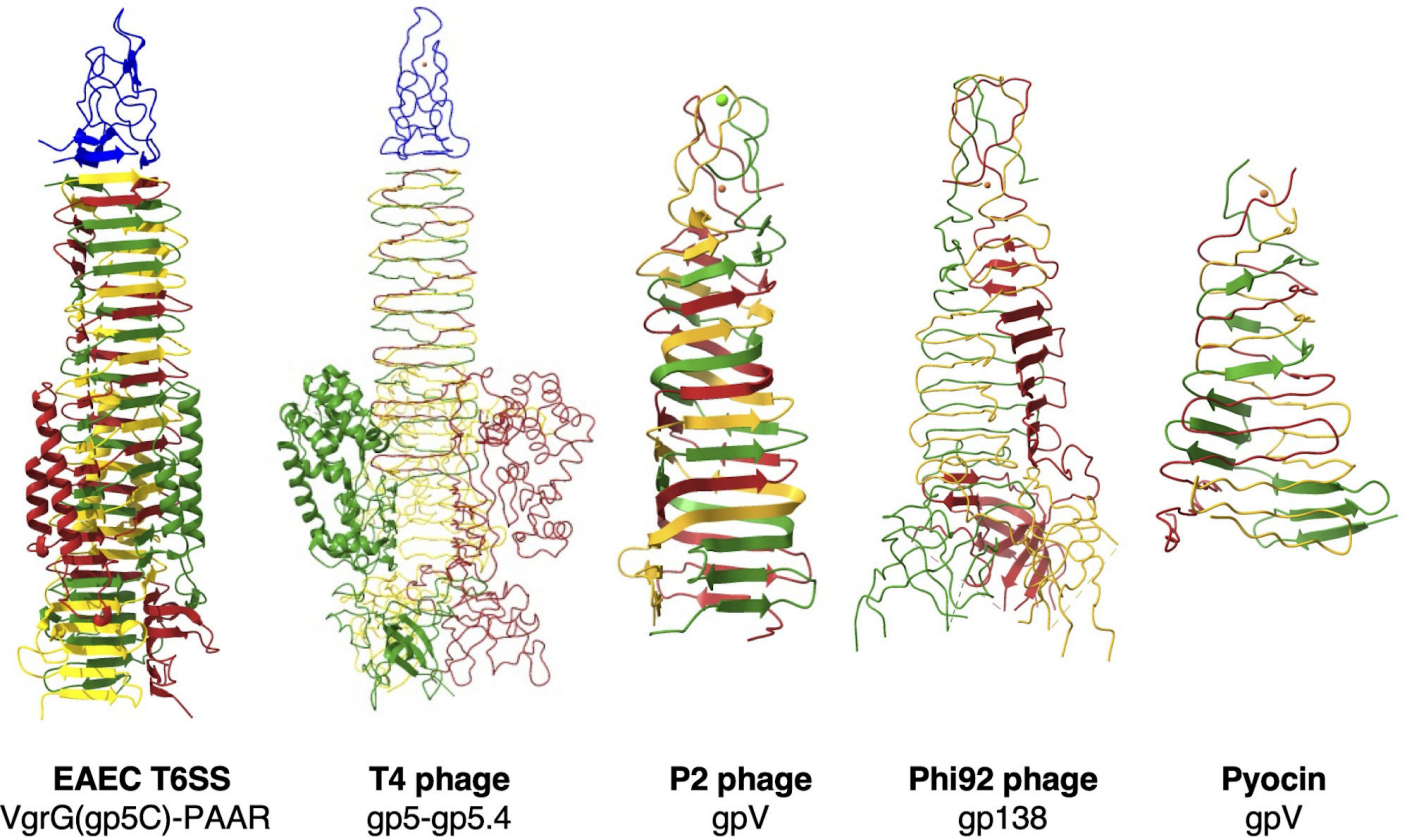
A conical tip structure consolidated by a metal atom is conserved in different phages and bacterial injection systems. Ribbon diagrams of VgrG(gp5C)-PAAR from EAEC, gp5-gp5.4 from T4 phage (PDB:5iv5), gpV from P2 phage (PDB:3aqj), gp138 from Phi92 phage (PDB:3pqi), and gpV from *R2* pyocin (PDB:4s36). All β-prism spikes are capped and the tip is closed with coordinated metal. Structures are colored by chain.

Ten years ago, Shneider and colleagues have first reported structures of a small protein PAAR that sharpens the T6SS spike (28). It was then proposed to stabilize the baseplate hub protein VgrG, by nucleating the folding of its trimers, or to regulate the incorporation of the VgrG into the T6SS machinery (28). However, we have found that the steady-state levels of VgrG^EAEC^ protein are not significantly different without the PAAR^EAEC^, and that the overproduction of VgrG^EAEC^ in the absence of PAAR^EAEC^ does not restore the T6SS-1 assembly and activity (Fig. S5 and Fig. S6). Similarly, the intracellular level of VgrG2 is not impaired in the absence of PAAR proteins in *Serratia marcescens* (30). Nevertheless, we have previously observed that purified VgrG^EAEC^ without PAAR^EAEC^ tends to precipitate in some buffers (21), and therefore, we cannot disregard the idea that PAAR could facilitate the folding of the VgrG trimer or its incorporation into the baseplate (28). It is important to mention that previous PAAR structures were obtained in chimeric complexes that were prepared using a soluble fragment of T4 gp5 β-helix and grafting the terminal β-helices of various VgrG spikes. Our attempts to crystallize PAAR from EAEC, alone or in complex with the gp5C part of VgrG, were unsuccessful. Nevertheless, our protein interaction studies supported by cryo-EM density maps docked with AlphaFold2 model presented in this study provide ultimate proof of the localization of PAAR protein at the top of the β-prism of the gp5 domain of VgrG (Fig. 1). The spike complex of the bacteriophage T4 adopts highly similar structure where a small conical gp5.4 protein sits on top of the β-prim of the gp5C (12). Strikingly, similar structures found in other phages such as P2 or Phi92 sharpen the apex of their needle by tying the ends of the gp5 β-helix into the knot that overall resembles the conical PAAR protein (Fig. 6) (35). Moreover, all these needle end structures are supported by the coordinated metal at the far end of the sharp structures (Fig. 6). We unambiguously showed that the purified PAAR^EAEC^ protein contains zinc. At least two conserved cysteines and one histidine, and likely a second histidine, are involved in the binding of this zinc atom. Their essentiality *in vivo* (C41, C74, and H46; Fig. 3 and Fig. 4) supports the crucial role of metal coordinated tip structure in the T6SS function. While the AlphaFold2 model of PAAR shows histidine residues oriented slightly outward, it is likely that they constitute the coordination center for the zinc atom and are hence pulled inwards to yield compact and sharp tip (Fig. 2). Similarly, three histidine and one cysteine residues were shown to be involved in zinc binding in the homologous PAAR proteins from *V. cholerae* or *E. coli* CFT073 and in the gp5.4 protein of phage T4 (12, 28). In the case of *Francisella* PAAR-like T6SS tip protein IglG, four cysteines were shown to be important for binding of iron or zinc (31). Interestingly, the apex domains of gp138 of Phi92 phage, gpV of P2 phage, and gpV of the *R2* pyocin all contain iron, hexacoordinated by six histidines symmetrically provided by three monomers [Fig. 6, PDB:4s3 (35, 36)]. In the light of these observations, our results further support the role of metal coordinating tip structures in sharpening spikes of membrane attacking contractile systems.

While the sharp tip might ensure the efficient piercing of the membrane, it does not readily explain the requirement of the PAAR for the assembly of T6SS^EAEC^. PAAR could be required for the recruitment of the spike to the membrane complex. In this case, the sharp tip maintained by the zinc atom may be important for this function. Interestingly, expression of the VgrG^EAEC^-Tle1^EAEC^ complex yielded virtually all spike complexes stuck head-to-head through the hydrophobic blunt end of the VgrG β-prisms as observed by cryo-EM (Fig. 1D; Fig. S1B). These interactions involve the hydrophobic patch otherwise covered by PAAR [(21), Fig. 1; Fig. S1B]. Indeed, the presence of PAAR^EAEC^ seems to prevent this aberrant behavior of VgrG^EAEC^ (Fig. 1D and E; Fig. S1A). Strikingly, the same head-to-head interaction occurs in the case of T4 phage gp5 needle when produced alone. Without the PAAR homolog gp5.4, the β- prism of gp5 dimerizes through its C-terminal hydrophobic tips and these dimers of trimers can form into two different orientations (37). We could also observe different 2D classes of twin VgrG^EAEC^-Tle1^EAEC^ complexes likely representing continuous or 60° rotated interactions of β-prisms (Fig. S1B). These observations further suggest that the amyloid-like structure of gp5 domain must be concluded to achieve a closed sharp end. Indeed, formation of a baseplate and its attachment to the membrane complex could be corrupted in the case of self-dimerization of VgrG^EAEC^ trimers. Moreover, this proposed role of PAAR protein in T6SS assembly could explain previous results showing that gp27 and gp5-OB domain of VgrG are sufficient for T6SS assembly and firing (20, 38). Indeed, without its β-prism, the needle-less trimeric VgrG may not dimerize this way and thus PAAR may not be required for T6SS assembly in this case.

Of note, despite the addition of PAAR protein, no interpretable density was again observed for the base of VgrG corresponding to the gp27-gp5 OB fold-like domains (21). Only one cryo-EM 2D class of particles displayed a distinct triangular shape structure at the base of the needle, suggesting that the flexibility of this domain is not stabilized by the presence of PAAR (Fig. S1A and B ). On the contrary, in the presence of PAAR, most of the cryo-EM 2D classes were missing densities for one, two, or three Tle1 toxins. Closer observations revealed that the EM densities around the middle of the gp5 structure were frequently visible and overall more intense. The missing toxin densities are therefore likely a result of the flexibility. It is possible that the head-to-head dimer of VgrG-Tle1 fixes the Tle1 toxins in a more rigid conformation than the VgrG-Tle1-PAAR complex. In particular, the TTR domain of VgrG that is hanging on a flexible linker might be less constrained in the presence of PAAR than in the head-to-head VgrG-Tle1 dimer. Gel filtrations consistently show uniform peak for VgrG-Tle1-PAAR complex and Tle1 seems to be in a stoichiometric equilibrium with VgrG based on band intensities in SDS-PAGE gels (Fig S7). This suggests that Tle1 toxins are efficiently loaded on the spike but are likely more flexible than previously observed. This flexibility might play an important role when encountering the target and liberating the toxin from spike.

To conclude, we suggest that while the overall large structure of the T6SS firing tube is built on the hexameric symmetry, it has to be resolved in order to be sealed and sharpened. First, symmetry transition is enabled by the VgrG-trimer that starts with pseudo-hexameric symmetry at its base and wraps it up to a trimeric β-prism. Furthermore, PAAR protein initiates by a pseudo-trimeric symmetry that connects to the trimeric β-prism. Toward the extremity, PAAR is sealed into a sharp end with the help of a metal coordinated by conserved cysteines and histidines. The importance of such architecture is supported by the fact that the closure of the puncturing tip with the help of metal coordinating center is structurally conserved across contractile injection systems that carry gp5-like β-prism domains (12, 28, 31, 35, 36). In some, but not all cases, the final conical tip is encoded as a separate protein. One has to take into account that in the case of T6SS, such separation provides an additional anchoring point for toxins. Hence, the separate open reading frame coding for the tip of puncturing device might have been evolutionary favored. Nevertheless, as demonstrated in this work, even without carrying toxic domains, sharpening the T6SS tip by PAAR protein is essential for the assembly and activity of the T6SS machinery in EAEC.

## MATERIALS AND METHODS

### Bacterial strains, growth conditions, and chemicals

Strains used in this study are listed in Table S1. *Escherichia coli* DH5, BL21(DE3), and W3110 were used for cloning procedures, protein production, and as a prey for antibacterial competition assays, respectively. EAEC strain 17-2 and its isogenic derivatives were used for functional studies. Cells were grown at 37°C with aeration in lysogeny broth (LB) with antibiotics when required [ampicillin (100 µg/mL), kanamycin (50 µg/mL), or chloramphenicol (30 µg/mL)] or in T6SS-1-inducing medium (SIM; M9 minimal medium, 0.2% glycerol, 1 µg/mL vitamin B1, 100 µg/mL casamino acids, 10% LB, supplemented or not with 1.5% Bacto agar) (39). Gene expression was induced by the addition of 0.02%–0.2% of L-arabinose (Sigma-Aldrich) for pBAD (40) and by 0.5 mM or 1 mM of isopropyl-thio-β-D-galactopyranoside (IPTG; Eurobio) for pETDuet-1, pRSFDuet-1, and pACYCDuet-1 (Novagen) derivative vectors.

### Cloning procedures, plasmid, and strain constructions

The plasmids and primers (obtained from Merck or Integrated DNA Technologies) used in this study are listed in Table S1. Cloning was performed by standard restriction-ligation procedures—DNA fragments coding for PAAR and VgrG were amplified from EAEC 17-2 chromosomal DNA using Q5 high-fidelity DNA polymerase (New England Biolabs), and PCR fragments were purified on NucleoSpin Gel and PCR Clean-up columns (Macherey-Nagel), digested as recommended by the manufacturer (New England Biolabs), and purified before ligation. Recombinant plasmids were transformed into *E. coli* DH5α cells and constructions were verified by colony PCR and by DNA sequencing (Eurofins) after extraction of the plasmids using Wizard Plus SV Minipreps kit (Promega).

For pACYC-PAAR^FL^ construct, the sequence encoding PAAR was amplified by PCR using 5-NdeI-PAAR and 3-XhoI-PAAR^FL^ primers introducing NdeI and XhoI sites, respectively, and cloned into the pACYCDuet-1 (Novagen) multiple cloning site 2. The 3- XhoI -PAAR^FL^ primer introduces a sequence encoding a FLAG-tag to allow in-frame fusion of PAAR with a C-terminal FLAG extension. For pETDuet-^H-SUMO^-PAAR construct, the sequence encoding PAAR was amplified by PCR using primers 5-BmtI-PAAR and 3-HindIII-PAAR introducing BmtI and HindIII sites, respectively, and cloned into the pETDuet-HIS-SUMO multiple cloning site.

The pBAD33-PAAR^VSVG^ was constructed by megapriming. The 3pBAD-4537-VSVG primer introduces a sequence encoding a VSV-G-tag to allow in-frame fusion of PAAR with a C-terminal VSV-G extension (VSVG). Site-directed mutagenesis of *PAAR* was performed on pACYC-PAAR^FLAG^, pETDuet-^H-SUMO-^PAAR, or pBAD33-PAAR^VSVG^ by QuickChange PCR-based targeted mutagenesis using complementary pairs of oligonucleotides and the PfuTurbo high-fidelity polymerase (Agilent Technologies).

For strain construction, the *PAAR* gene (EC042_4537) was deleted into the EAEC 17-2 wild-type strain, the *tssB-sfGFP*, the *tssK-sfGFP*, and the *sfGFP-tssM* using a modified one-step inactivation procedure (41) as previously described (42) using pKD4 plasmid and oligonucleotide pairs DEL-4537-5-DW/DEL-4537-3-DW. Kanamycin-resistant clones were selected and verified by colony PCR and sequencing of the region (Eurofins genomics).

### SDS-PAGE and Western blot analyzes

SDS-PAGE was performed using standard protocols. Proteins were stained using Instant*Blue* (Sigma-Aldrich) or transferred onto 0.2 μm nitrocellulose membrane (Amersham Protran). Immunoblots were probed with anti-Strep-Tag Classic (clone Strep-tag II, Bio-Rad catalog #MCA2489), anti-FLAG (clone M2, Sigma-Aldrich catalog #F3165), anti-His-Tag (clone 1B7G5, Proteintech catalog #66005–1-Ig), anti-VSV-G (clone P5D4; Sigma-Aldrich) or anti-RecA (clone M2, Sigma-Aldrich catalog #F3165) primary monoclonal antibodies, and alkaline phosphatase-conjugated goat anti-mouse secondary antibodies, and revealed in alkaline buffer (pH 9) using 5-bromo-4-chloro-3-indolyl phosphate/nitroblue tetrazolium in the presence of 10 mM MgCl_2_.

### Protein production and pull-down assays

An overnight culture of *E. coli* BL21(DE3) co-transformed with pACYC-PAAR^FL^ (or pACYC-PAAR^FL^ derivatives) and pET-^S^VgrG (or pET-^S^VgrG derivatives) was diluted 1/100 into 50 mL of LB supplemented with the required antibiotics, grown to *A*_600_ =0.8, and induced with 1 mM IPTG for 18 h at 16°C. Cells were pelleted by centrifugation at 5,000 × *g*, and resuspended in buffer A [50 mM Tris–HCl pH 8.5, 150 mM NaCl, 1 mM Tris(2-carboxyethyl)phosphine hydrochloride (TCEP), complete EDTA-free protease inhibitor cocktail (Sigma)]. After cell lysis by sonication, the cell extract was cleared by centrifugation for 45 min at 15,000 *× g* and incubated for 1 h at 4°C with gentle mixing with 100 µL of Strep-Tactin Superflow resin (IBA Technology) previously equilibrated in buffer A. The resin was washed five times with 300 µL of buffer A and the ^S^VgrG-PAAR^FL^ was eluted with 100 µL of buffer B (50 mM Tris–HCl pH 8.5, 150 mM NaCl, 1 mM TCEP, 2.5 mM desthiobiotin). Ten microliters of the elution fractions was separated by 12.5% acrylamide SDS-PAGE, stained using Instant*Blue* (Sigma-Aldrich), or analyzed by Western blot with anti-Strep-Tag Classic or anti-FLAG antibodies.

For the production of Tle1-VgrG-PAAR complex, BL21(DE3) was co-transformed with pRSF-Tle1^H^, pET-^S^VgrG, and pACYC-PAAR^FL^ plasmids. ^S^VgrG-Tle1^H^-PAAR^FL^ complex was then produced and purified using the same double-step affinity procedure as for the purification of the ^S^VgrG-Tle1^H^ complex described in reference (21).

### Cryo-EM sample preparation, data collection, and modeling

Quantifoil Cu300 mesh *R2*/2 grids were glow discharged at 0.2 mbar vacuum, 2mA current for 45 s (in an ELMO glow discharge system (Cordouan Technologies). Four microliters of protein samples at concentration of 0.5 mL/mL were blotted and plunge-frozen in liquid ethane using Vitrobot instrument (Field Electron and Ion Company [FEI]) at 4°C and 100% humidity, using blot force 0 and blotting time 3 s. Grids were transferred to liquid nitrogen for storage. Observation and data were collected with Talos Arctica (FEI) 200 kV microscope equipped with K2 camera (Gatan). Data were collected at 45 k magnification, pixel size of 0.93 Å, with 4.4 s exposition and total dose of 50 e-/Å with the help of SerialEM version 3.8 software. Two thousand one hundred eighty movies were collected for VgrG-Tle1 complex (without PAAR) data set. Three thousand two hundred thirty-seven movies were collected for VgrG-Tle1-PAAR data set. Data were processed with CryoSPARC v4.0.0. Particle picking was assisted by four iterations of Topaz (43) training for 20 epochs, each time resubmitting best quality particles for new iteration of training. For VgrG-Tle1 (without PAAR) data set: 256,139 particles were extracted with 550 pixels box size. For VgrG-Tle1-PAAR data set: 436,572 particles were extracted with 400 pixels box size. Best-defined 2D classes were used for generation four *ab initio* models, and best models were refined using heterologous refinement. For high-resolution VgrG-Tle1-PAAR map, particles used for best class refinement were again used to generate four *ab initio* models and best model was then refined using best-defined 2D classes. Final step of non-uniform refinement with 31,647 particles was used to gain resolution in best regions.

VgrG-Tle1-PAAR model was generated as follows: gp5 domain of VgrG (three copies) and PAAR were co-folded using AlphaFold2 (44, 45). Using UCSF ChimeraX (46), the best ranked AlphaFold2 model was aligned to VgrG-Tle1 model [PDB:6SJL, (21)] via the gp5 domains. This model was then fitted into cryo-EM density maps (46).

### His-SUMO-PAAR purification and samples preparation for ICP-OES

Overnight cultures of *E. coli* BL21(DE3) transformed with pETDuet-^H^SUMO-PAAR or its mutated derivatives were diluted 1/100 into 2L of LB supplemented with the required antibiotics. Cells were grown to an *A*_600_ =0.8 and induced with 1 mM IPTG for 18 h at 16°C. Cells were pelleted by centrifugation at 5,000 *× g*, resuspended in buffer C [20 mM HEPES pH 8, 250 mM NaCl, 1 mM TCEP, complete EDTA-free protease inhibitor cocktail (Sigma) 100 µg/mL DNase I, 100 µg/mL lysozyme, and 10 mM MgCl_2_]. After cell lysis using an EmulsiFlex-C5 (Avestin), the cell extract was cleared by centrifugation for 40 min at 40,000 × *g* and incubated for 1 h at 4°C with gentle mixing with 1 mL of Strep-Tactin Superflow resin (IBA Technology) previously equilibrated in buffer C. The resin was washed five times with 8 mL of buffer C and the ^H-^SUMO-PAAR was eluted with 100 µL of buffer D (20 mM HEPES pH 8, 250 mM NaCl, 1 mM TCEP, 500 mM imidazole). Ten microliters of the elution fractions was separated by 15% acrylamide SDS-PAGE, stained using Instant*Blue*, or transferred onto nitrocellulose membrane and immunodetected with anti-His-Tag antibodies.

### Metal quantification by inductively coupled plasma optical emission spectrometry

Fifty to 300 µM of proteins in 500 µL of phosphate-buffered saline (PBS) were diluted in 500 µL of 69% nitric acid. Samples were boiled for 40 min and 4 mL of 3% nitric acid was added to each sample. These samples were analyzed for copper, nickel, zinc, and iron content with an iCAP 6000 series optical emission spectrometer (Thermo Scientific). Serial dilutions of pure copper, nickel, iron, and zinc standard solutions were used for calibration. Results are expressed in percentage of total proteins in samples, each value representing the mean of three technical replicates from different fractions of two to three different purification preparations. As a control, 500 µL of purification buffer was submitted to the same metal content analysis. For statistical analysis, one-way analysis of variance (ANOVA) followed by Dunnett’s multiple comparisons test was performed using GraphPad Prism version 9.3.1 for macOS (GraphPad Software, San Diego, CA, USA).

### Fluorescence microscopy

Overnight cultures grown in LB medium with the appropriate antibiotics were diluted 1/100 into SIM to an *A*_600_ = 0.8, then 500 µL of the culture was centrifuged and resuspended in fresh SIM to *A*_600_ = 10. Cells were spotted on a thin 2% agarose pad, prepared with SIM medium in Frame-Seal slide chambers (Bio-Rad), and covered with a cover slip. After 20 min at 28°C, Hilo fluorescence microscopy was performed with a Nikon Eclipse Ti2 microscope equipped with a 100× numerical aperture 1.45 Ph3 objective, an Orca-Fusion digital camera (Hamamatsu), a perfect focus system, and an Ilas2 total internal reflection fluorescence (TIRF)/fluorescence recovery after photobleaching (FRAP) module (Gataca Systems). Fluorescence images were acquired with an exposure time of 100 ms for phase contrast, sfGFP-TssM, TssK-sfGFP, and 50 ms for TssB- sfGFP. The experiments were conducted in triplicate and a representative result is shown. The microscopy images were analyzed using ImageJ (http://imagej.nih.gov/ij/).

### Antibacterial competition assay by the colorimetric method

Overnight cultures grown in LB medium with the appropriate antibiotics were diluted 1/100 into SIM medium to an *A*_600_ = 0.4. Then, 50 µM of IPTG was added in the culture of W3110 bacterial prey cells to induce the production of β-galactosidase. WT or mutated *PAAR* genes expression was induced from pBAD33-*PAAR^VSVG^* using 0.2% arabinose. At *A*_600_ = 0.8, the predator and the prey cultures were mixed at 1:4 ratio and spotted on SIM agar plates supplemented with 50 µM of IPTG and 0.2% arabinose. After 2 h of incubation at 37°C, drops of 2 mM CPRG solution were deposited on the cell spots. Pictures were taken after the emergence of the coloration. Experiment was performed at least three times and a representative image is presented. For quantification of CPR, slices of agar-containing spots were vortexed in 500 µL PBS and further centrifuged at 8,000 × *g* for 1 min to pellet cells and agar. One hundred microliters of the supernatant was dropped into a 96-well plate. After addition of 10 µL of 2 mM CPRG into the supernatant, absorbance at λ = 572 was measured every 30 s for 1 h with a microplate reader (TECAN).

### Antibacterial competition assay by the emergence time method

Overnight cultures grown in LB medium with the appropriate antibiotics were diluted 1/100 into SIM medium to an *A*_600_ = 0.4. Arabinose 0.2% was added into predator strain cultures to induce *PAAR* gene expression from pBAD33-*PAAR^VSVG^* (or pBAD33-*PAAR^VSVG^* mutants). At *A*_600_ = 0.8, the predator strain and the prey were mixed at 4:1 ratio and spotted on SIM agar plates supplemented with 0.2% arabinose. After 4 h of incubation at 37°C, each spot was resuspended in 1 mL of selective medium for prey and 100 µL of a 10-fold dilution was added to 100 µL of selective medium in 96-well microplate (Thermo Fisher, Nunc). Cultures were grown for 15 h at 37°C with agitation and *A*_600_ was measured each 5 min using a microplate reader (TECAN). A time of emergence considered as the time required for each sample to reach *A*_600_ = 0.4 was calculated. Each value represents the mean of three technical replicates from at least three biological replicates and were compared between strains by a one-way ANOVA followed by Dunnett’s multiple comparisons test performed using GraphPad Prism (version 9.3.1 for macOS, GraphPad Software, San Diego, CA, USA). Negative controls contained only the prey or the predator strain. EAEC 17-2 wild-type strain was used as positive control for W3110 prey lysis.

Production in EAEC ΔPAARΩKan of the different derivatives of PAAR protein from pBAD plasmids used for antibacterial competition and fluorescence microscopy observations were controlled by Western blotting using anti-VSV-G antibodies.

### Protein stability assay

Overnight cultures of *E. coli* DH5α transformed with pBAD18-PAAR^VSVG^ and pOK-VgrG^FLAG^ or empty pBAD18 and pOK-VgrG^FLAG^ were diluted 1/100 into 50 mL of LB medium supplemented with the required antibiotics, grown at 37°C to an *A*_600_ of 0.6 and induced with 0.5 mM IPTG and 0.2% arabinose. After 1 h, spectinomycin (100 µg/mL) and chloramphenicol (40 µg/mL) were added. Before and after the induction, at 1 h, 2 h, and 3 h after the inhibition of protein synthesis, 2 mL of cultures was collected, centrifuged for 5 min at 6,000 *× g,* and supplemented with 60 µL or 100 µL of 4× SDS-PAGE loading dye with β-mercaptoethanol. Proteins were separated by SDS-PAGE and transferred onto nitrocellulose membrane for immunodetection with anti-VSVG, or anti-FLAG or anti-RecA monoclonal antibodies, followed by recognition by secondary antibodies coupled to the alkaline phosphatase. Intensity of the bands was measured using BioRad Imager.

### Computer algorithms

EAEC PAAR orthologs (EC042_4537; NCBI protein ID: CBG37359) were retrieved using BlastP analysis against the Kyoto Encyclopedia of Genes and Genomes database and aligned with MultiAlin tool (47, 48).

## SUPPLEMENTAL MATERIAL

Supplemental material spectrum.01478-23-s0001.pdf Single pdf containing Fig. S1 to Fig. S7 and Table S1.

ASM does not own the copyrights to Supplemental Material that may be linked to, or accessed through, an article. The authors have granted ASM a non-exclusive, world-wide license to publish the Supplemental Material files. Please contact the corresponding author directly for reuse.
