# Supplementary material for: The tip protein PAAR is required for the function of the type VI secretion system
Source: Microbiol Spectr. 2023 Oct 6;11(6):e01478-23. doi: 10.1128/spectrum.01478-23 (PMC10715212; doi:10.1128/spectrum.01478-23)
Supplement: Supplemental material — Single pdf containing Fig. S1 to Fig. S7 and Table S1. [file spectrum.01478-23-s0001.pdf]

SUPPLEMENTAL MATERIAL

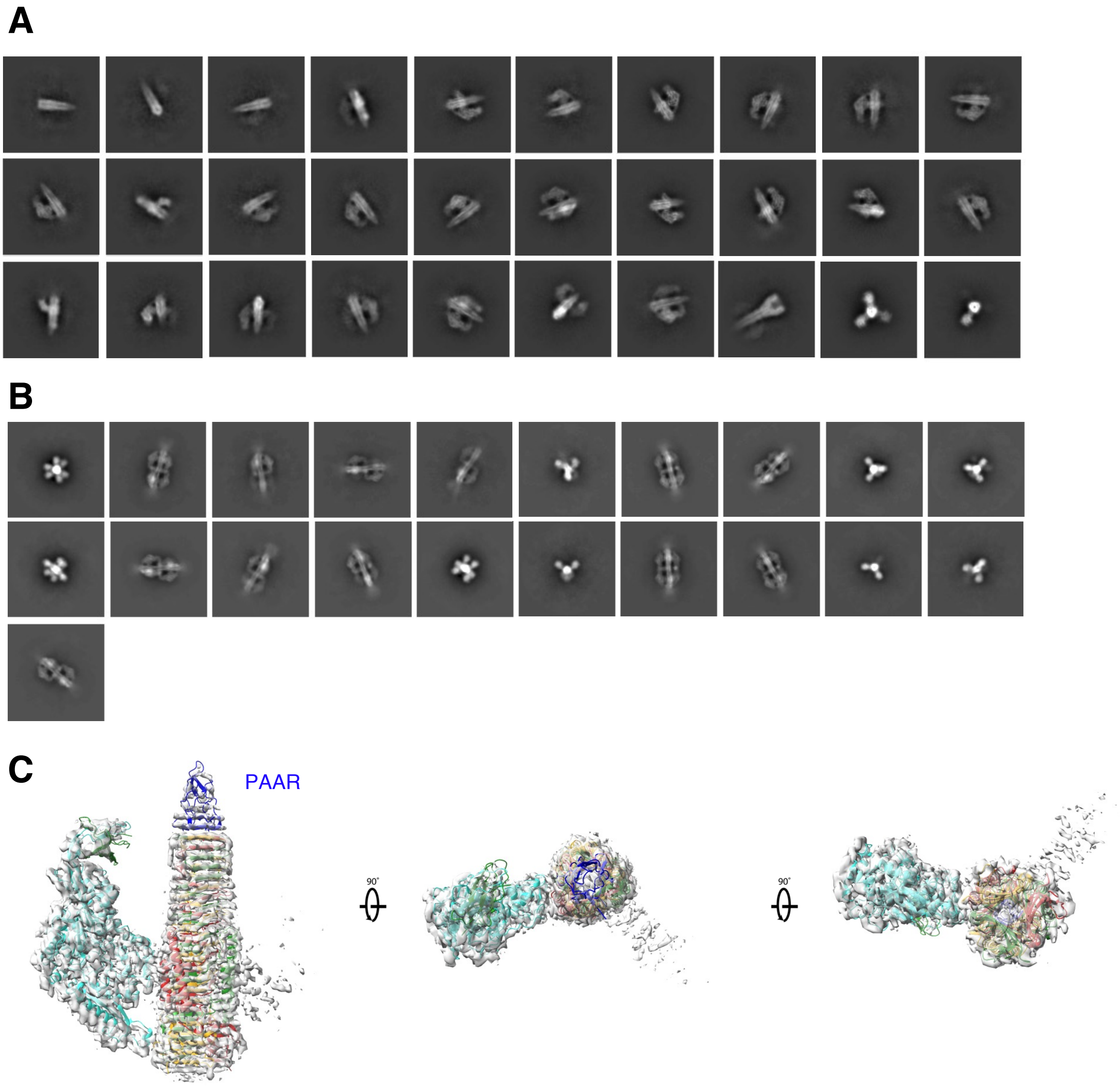

Fig. S1. 2D classes of VgrG-Tle1 and VgrG-Tle1-PAAR complexes.

(A) Representative 2D classes of VgrG-Tle1-PAAR complex in different orientations. No violin bodies double particles were observed, however most of the particles lack at least one Tle1 density. One particular class of highly structured VgrG base corresponding to a small fraction of particles (1%) was also observed. **(B)** Representative 2D classes of VgrG-Tle1 in different orientations. Most of the particles correspond to dimers of $(VgrG)_3$-$(Tle1)_3$ complexes. **(C)** VgrG-Tle1-PAAR complex model. Alphafold2 co-folding model of the needle domain of VgrG (3 copies, green, yellow, red) with PAAR (blue) was aligned with the VgrG-Tle1 model (PDB:6SJL) merging on the needle domain. This model was fitted in the cryo-EM density maps (3.7 Å, one Tle1 visible).

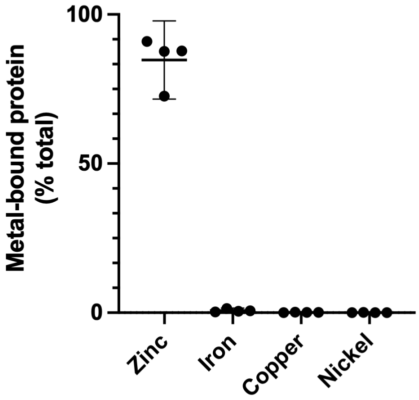

Fig. S2. Nor iron, nickel or copper were detected by ICP-OES in PAAR protein samples. Iron, nickel and copper content analysis using ICP-OES of purified His-SUMO-PAAR (WT). Each value represents the mean of three technical replicates from different fractions.

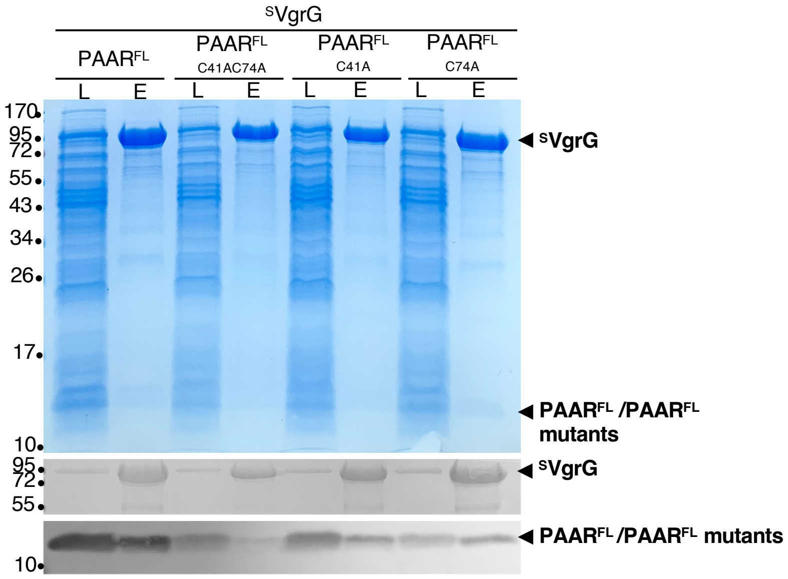

Fig. S3. PAAR mutants still interact with the VgrG needle domain.
Pull-down assays. Lysates (L) of BL21(DE3) cells co-producing Strep-tag II-tagged VgrG (^{S}VgrG) and FLAG-tagged PAAR (PAAR^{FL}) and or PAAR mutated versions (PAAR$^{C41AC74\text{-}FL}$, PAAR$^{C41A\text{-}FL}$ OR PAAR$^{C74A\text{-}FL}$) were loaded on a Strep-Tactin column. After washing steps, desthiobiotin-eluted (E) fractions were analyzed by SDS-PAGE and Coomassie blue staining (upper panel), immunoblot using anti-StrepII (middle panel) and anti-FLAG (lower panel).

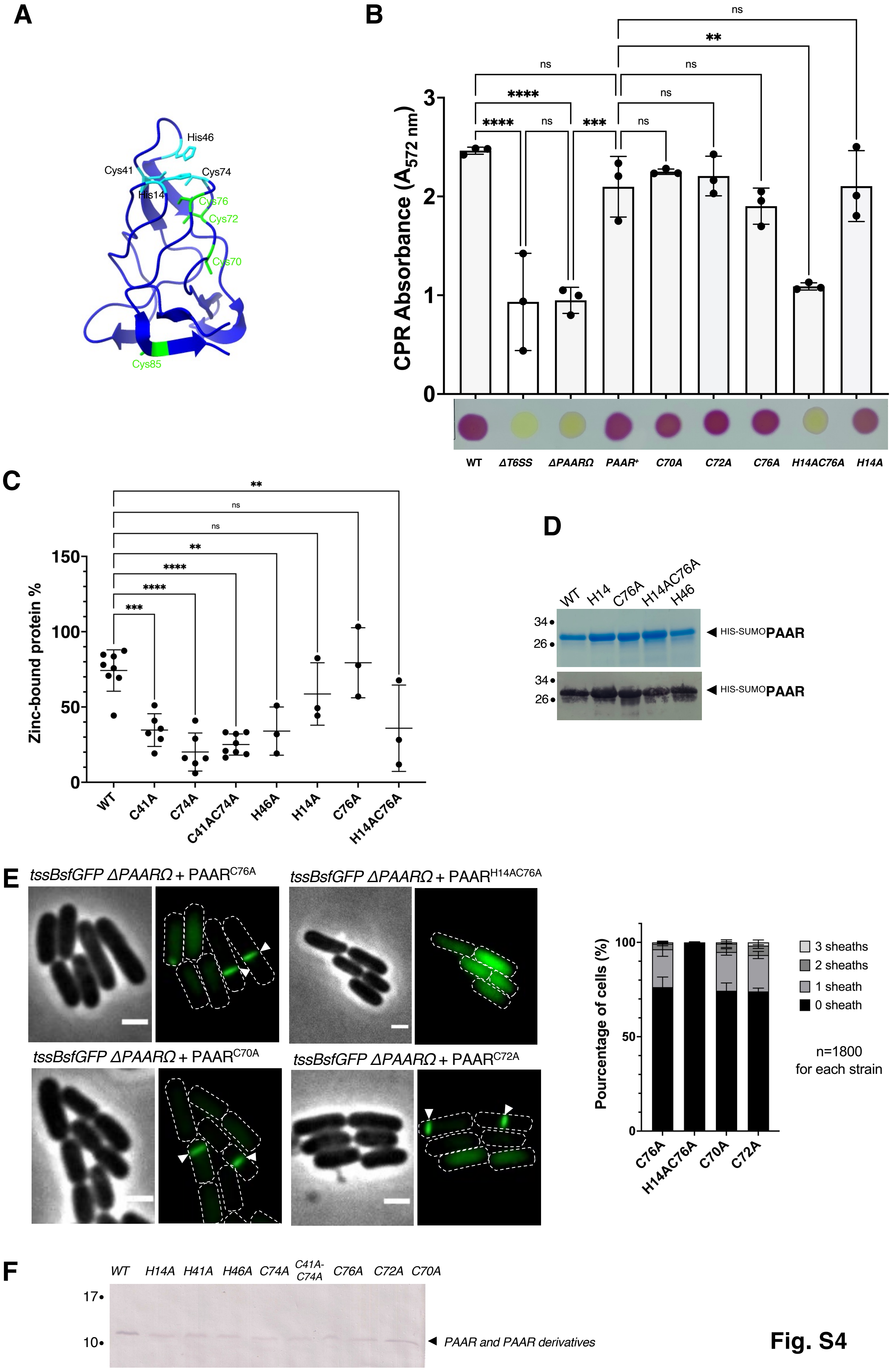

A
His46
Cys41
Cys74
His14
Cys76
Cys72
Cys70
Cys85
B
CPR Absorbance (A572 nm)
ns
**
ns
****
ns
****
ns
***
ns
0
1
2
3
WT
ΔT6SS
ΔPAARΩ
PAAR+
C70A
C72A
C76A
H14AC76A
H14A
C
Zinc-bound protein %
**
ns
ns
**
****
****
***
0
50
100
150
WT
C41A
C74A
C41AC74A
H46A
H14A
C76A
H14AC76A
D
WT
H14
C76A
H14AC76A
H46
34
26
HIS-SUMOPAAR
34
26
HIS-SUMOPAAR
E
tssBsfGFP ΔPAARΩ + PAARC76A
tssBsfGFP ΔPAARΩ + PAARH14AC76A
tssBsfGFP ΔPAARΩ + PAARC70A
tssBsfGFP ΔPAARΩ + PAARC72A
Pourcentage of cells (%)
0
50
100
C76A
H14AC76A
C70A
C72A
3 sheaths
2 sheaths
1 sheath
0 sheath
n=1800
for each strain
F
WT
H14A
H41A
H46A
C74A
C41A-
C74A
C76A
C72A
C70A
17
10
PAAR and PAAR derivatives

Fig. S4

Fig. S4. Cys70, Cys72 and Cys76 are not necessary for sheath assembly and T6SS-1 mediated killing.

(A) Alphafold model of $PAAR^{EAEC}$ protein highlighting zinc binding residues (in cyan) and other Cys and His residues (in green). **(B)** Antibacterial competition assay by the Colorimetric method. The T6SS-1 function of the WT, ∆T6SS, ΔPAAR**Ω** strains and ΔPAAR**Ω** strain complemented with PAAR or PAAR Cys and His mutant strains (using $pBAD_{33}$-$PAAR^{VSVG}$ and corresponding PAAR Cys and His mutant plasmids derivatives) was tested by assessing their ability to kill W3110 *E. coli* K12 bacterial prey. Killing efficiency was monitored by observing degradation of yellow CPRG into purple CPR by free ß-galactosidase released from lysed prey cells after being exposed to the predator cells. CPR absorbance (A_{572nm}, upper graph) was measured from the spots (lower panel). The means of three biological replicates are indicated. The error bars represent standard deviation. Statistical significance was calculated using Ordinary one-way ANOVA followed by Dunnett's multiple comparisons test using GraphPad Prism. **** p<0.0001; *** p=0.0002; ** p=0.0010; ns=0,6086 (WT vs. $PAAR^{+}$); ns=0.9980 ($PAAR^{+}$ vs. $PAAR^{C70A}$); ns=0.9999 ($PAAR^{+}$ vs. $PAAR^{C72A}$); ns=0.9860 ($PAAR^{+}$ vs. $PAAR^{C76A}$); ns>0.9999 (∆T6SS vs. ΔPAAR**Ω** and $PAAR^{+}$ vs. $PAAR^{H14A}$). **(C)** Zinc content analysis using ICP-OES of purified His-SUMO-PAAR (WT) and cysteine mutants. Statistical significance was calculated using Ordinary one-way ANOVA followed by Dunnett's multiple comparisons test using GraphPad Prism. **** p<0.0001; *** p=0.0002; ** p= 0.0025 (WT vs. H46A), **p= 0.0042 (WT vs. H14AC76A), ns=0.5483 (WT vs. H14A); ns= 0.9969 (WT vs. C76A). Each value represents the mean of three technical replicates of two to four different fractions of one (H14A, H46A, C76A, H14AC76A), two (C41A, C74A) to three different (WT, C41C74A) purifications preparations. **(D)** SDS-PAGE followed by Coomassie blue staining (upper panel) and immunoblot using anti-His-Tag (lower panel) of purified wild type His-SUMO-PAAR proteins and mutated versions (His-SUMO-$PAAR^{H14A}$, His-SUMO-$PAAR^{C76A}$ His-SUMO-$PAAR^{C14AC76A}$ and His-SUMO-$PAAR^{H46A}$). The molecular weight markers (in kDa) are indicated on the left. **(E)** Fluorescence microscopy of EAEC *tssB-sfGFP ΔPAAR ΩKan* strains transformed with plasmid $pBAD_{33}$-$PAAR_{C76A}$-^{VSVG}, pBAD33-$PAAR_{C72A}$-^{VSVG}, pBAD33-$PAAR_{C70A}$-^{VSVG} or pBAD33-$PAAR_{H14AC76A}$-^{VSVG}. Phase contrast images are shown on the left (scale bar = 2 µm). Fluorescence images of TssB-sfGFP (green) are shown on the right. Cells were outlined with white dashed lines, white arrows indicate dynamic sheath assembly and/or contraction events. Quantification of the number of extended sheaths per cell from the corresponding strains is presented on the graph. The total number of analyzed cells (n) from 3 independent biological replicates is indicated. The error bars represent standard deviation. **(F)** Immunodetection using anti-VSVG antibodies of the different versions of PAAR protein expressed from the corresponding $pBAD_{33}$ plasmids in ΔPAARΩ.

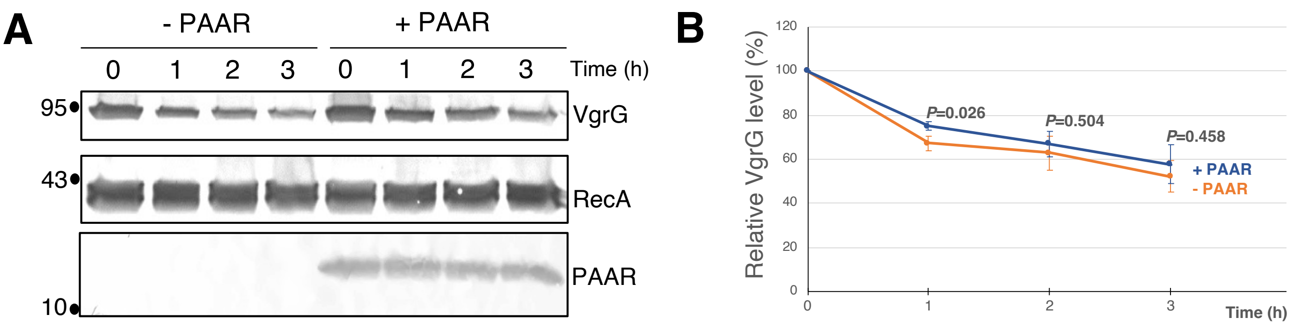

Fig. S5. Steady-state levels of VgrG in the presence or not of PAAR.
(A) VgrG protein stability over time after inhibition of protein synthesis at 0h. VgrG was produced in DH5α transformed with pOK-VgrG^{FLAG} and pBAD$_{18}$ empty (- PAAR) or pOK-VgrG^{FLAG} and pBAD$_{18}$-PAAR^{VSVG} (+ PAAR), and detected by Western Blot using anti-FLAG antibodies. Sample loading was controlled by detection of RecA protein on the same Western Blot. Production of PAAR^{VSVG} protein was controlled by Western Blot using anti-VSVG. Molecular weight standards (in kD) are indicated on the left, proteins detected using corresponding antibodies are indicated on the right. Time after inhibition of protein synthesis is indicated at the top in hours. **(B**) VgrG protein level at each timepoint was quantified by measuring bands intensities using a BioRad Imager and software. Intensity values were normalized to those of VgrG at 0h, considered as the initial quantity of protein (100%). Each value represents the mean (+/-SD) of 3 independent experiments. *P* values obtained from unpaired t-test are indicated (* p< 0.05, ns p>0.05). No statistically significant differences were observed. Blue line, + PAAR; orange line, - PAAR.

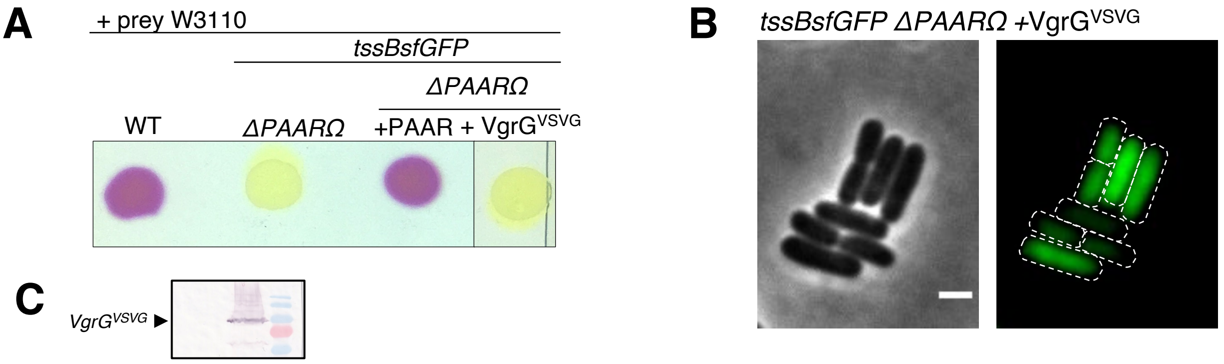

Fig. S6. Overproduction of VgrG in a *ΔPAARΩ* mutant does not restore the T6SS-1 function.
(A) Antibacterial competition assay by the *Colorimetric method.* The T6SS-1 function of the WT, *tssB-sfGFP ΔPAARΩKan* strains and *tssB-sfGFP ΔPAARΩKan* strain complemented with pBAD$_{33}$-PAAR^{VSVG} or pBAD$_{33}$-VgrG^{VSVG}, was tested by assessing their ability to kill W3110 *E. coli* K12 bacterial prey. Killing efficiency was monitored by observing degradation of yellow CPRG into purple CPR by free ß-galactosidase released from lysed prey cells after being exposed to predator cells. **(B)** Fluorescence microscopy of EAEC *tssB-sfGFP ΔPAARΩKan* strain transformed with plasmid pBAD$_{33}$-VgrG^{VSVG}. Phase contrast image is shown on the left (scale bar 2 µm). Fluorescence image of TssB-sfGFP (green) is shown on the right, cells were outlined with white dashed lines. **(C)** Immuno-detection of VgrG^{VSVG} production in EAEC *tssB-sfGFP ΔPAARΩKan* and *tssB-sfGFP ΔPAARΩKan* + pBAD$_{33}$-VgrG^{VSVG} strains used for fluorescence microscopy for **B**.

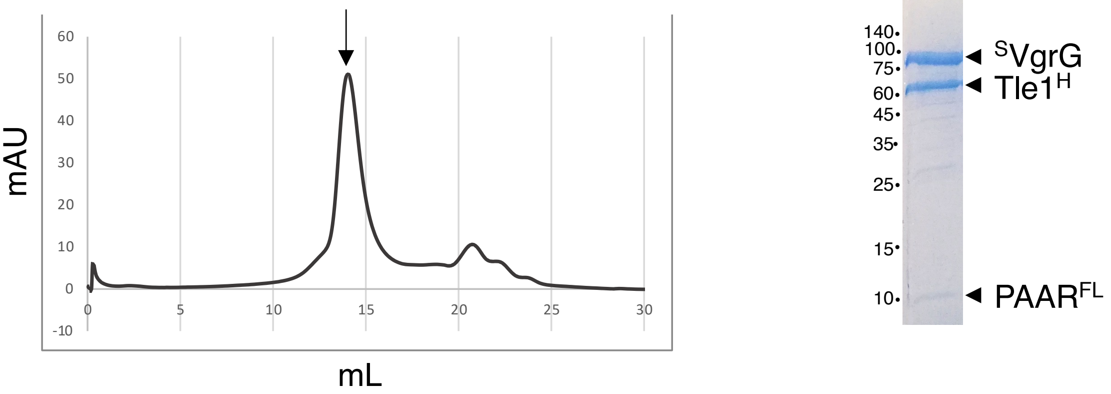

Fig. S7. Size-exclusion chromatography analysis of the purified ^{S}VgrG-Tle1^{H}-PAAR^{FL} complex. Gel filtration analysis was performed on a Superose 6 column. The position of the peak fractions corresponding to ^{S}VgrG-Tle1^{H-}-PAAR^{FL} is indicated at the top by an arrow. Ten μL of the fraction corresponding to the center of the peak were loaded on SDS-PAGE followed by Coomassie blue staining. Molecular weight markers in kDa and the positions of ^{S}VgrG, Tle1^{H} and PAAR^{FL} are indicated on the left and right, respectively.

Table S1. Strains, plasmids and oligonucleotides used in this study.

Strains

**Strains**	**Description and genotype**	**Source**
Enteroaggregative *E. coli*		
17-2	Wild-type enteroaggregative *Escherichia coli*	Arlette Darfeuille-Michaud
17-2 Δ*PAARΩKan*	17-2 deleted of the T6SS-1 *PAAR* gene replaced by a kanamycin cassette, Kan^R	This study
17-2 *tssB-sfGFP*	Insertion of the sfGFP coding sequence upstream the stop codon of *tssB* in 17-2	(1)
17-2 *tssB-sfGFP* Δ*PAARΩKan*	17-2 *tssB-sfGFP* deleted of the T6SS-1 *PAAR* gene replaced by a kanamycin cassette, Kan^R	This study
17-2 Δ*T6SS-1*	17-2 deleted of the *sci-1* gene cluster	(2)
17-2 tssK-sfGFP	Insertion of the sfGFP coding sequence upstream the stop codon of *tssK* in 17-2	(3)
17-2 *tssK-sfGFP ΔPAAR ΩKan*	17-2 *tssK-sfGFP* deleted of the T6SS-1 *PAAR* gene replaced by a kanamycin cassette	This study
17-2 sfGFP-tssM	Insertion of the sfGFP coding sequence downstream the start codon of *tssM* in 17-2	(4)
17-2 *sfGFP-tssM* Δ*PAARΩKan*	17-2 *sfGFP-tssM* deleted of the T6SS-1 *PAAR* gene replaced by a kanamycin cassette	This study
E. coli K12		
DH5α	F-, Δ(*argF*-l*ac*)U169, *phoA*, s*upE44*, Δ(lac*Z*)M15, *relA*, *endA*, *thi*, *hsdR*	Laboratory collection
W3110	F-, lambda-IN(*rrnD-rrnE*)1 *rph*-1	Laboratory collection
BL21(DE3)	*fhuA2* [*lon*] *ompT gal* (λ DE3) [*dcm*] Δ*hsdS* λ DE3 = λ s*BamHI*oΔ*EcoRI*-B int::(l*acI*::PlacUV5::T7 gene1) i21 Δ*nin5*	Invitrogen

Plasmids

Vectors	Description	Source
pBAD33	pACYC-184 *ori*, p*Ara*, CmR	(5)
pBAD18	pBR322 *ori*, p*Ara*, Amp^R	(5)
pETDuet-1	Expression vector, *lacI*, *pT7*, AmpR	Novagen
pACYCDuet-1	Expression vector, *lacI*, *pT7*, CmR	Novagen
pET-^{S}VgrG	T6SS-1 *vgrG*, cloned into pET-Duet-1, N-terminal StrepTagII	(6)
pETDuet-^{S}VgrGΔTTR	T6SS-1 *vgrG* deleted of sequence encoding amino-acids 779 to 841, N-terminal StrepTagII	This study
pET-^{S}VgrG1-490 (ΔNeedle)	T6SS-1 *vgrG* deleted of sequence encoding amino-acids 491 to 841, N-terminal StrepTagII	(6)
pRSF-Tle1^H	T6SS-1 *tle1* cloned into pRSFDuet-1, 6×His C-terminal tag	(6)
pACYC-PAAR^{FLAG}	T6SS-1 *PAAR* cloned into pACYCDuet-1, C-terminal FLAG epitope	This study
pACYC-PAARC41A^{FLAG}	Cys41-to-Ala substitution introduced into pACYC-PAAR^{FLAG}	This study
pACYC-PAARC74A^{FLAG}	Cys74-to-Ala substitution introduced into pACYC-PAAR^{FLAG}	This study
pACYC-PAAR$^{C41AC74A\text{-}FLAG}$	Cys41-to-Ala and Cys74-to-Ala substitution introduced into pACYC-PAAR^{FLAG}	This study
pACYC-PAAR$^{H46A\text{-}FLAG}$	His46-to-Ala and Cys74-to-Ala substitution introduced into pACYC-PAAR^{FLAG}	This study
pACYC-PAAR$^{H14A\text{-}FLAG}$	His14-to-Ala and Cys74-to-Ala substitution introduced into pACYC -PAAR^{FLAG}	This study
pETDuet-$^{H\text{-}SUMO\text{-}}$PAAR	T6SS-1 *PAAR* cloned into pETDuet-1, N-terminal fusion to SUMO and 6*His tagged	This study
pETDuet-$^{H\text{-}SUMO\text{-}}$PAAR^{C41AC74A}	Cys41-to-Ala and Cys74-to-Ala substitution introduced into pETDuet-$^{H\text{-}SUMO\text{-}}$PAAR	This study
pETDuet-$^{H\text{-}SUMO\text{-}}$PAAR^{C41A}	Cys41-to-Ala substitution introduced into pETDuet-$^{H\text{-}SUMO\text{-}}$PAAR	This study
pETDuet-$^{H\text{-}SUMO\text{-}}$PAAR^{C74A}	Cys74-to-Ala substitution introduced into pETDuet-$^{H\text{-}SUMO\text{-}}$PAAR	This study
pBAD33-PAAR	T6SS-1 *PAAR* cloned into pBAD33	This study
pBAD33-PAAR^{VSVG}	T6SS-1 *PAAR* cloned into pBAD33, C-terminal VSVG epitope	This study
pBAD33-PAAR$_{C41A}$$^{-VSVG}$	Cys41-to-Ala substitution introduced into pBAD33-PAAR^{VSVG}	This study
pBAD33 -PAAR$_{C74A}$$^{-VSVG}$	Cys74-to-Ala substitution introduced into pBAD33-PAAR^{VSVG}	This study
pBAD33-PAAR$_{C41AC74A}$$^{-VSVG}$	Cys41-to-Ala and Cys74-to-Ala substitution introduced into pBAD33-PAAR^{VSVG}	This study
pBAD33-PAAR$_{H46A}$$^{-VSVG}$	His46-to-Ala substitution introduced into pBAD33-PAAR^{VSVG}	This study

pBAD33 $PAAR_{H14A}{}^{-VSVG}$	His14-to-Ala substitution introduced into pBAD33-$PAAR^{VSVG}$	This study
pBAD33 $PAAR_{C70A}{}^{-VSVG}$	Cys70-to-Ala substitution introduced into pBAD33-$PAAR^{VSVG}$	This study
pBAD33 $PAAR_{C72A}{}^{-VSVG}$	Cys72-to-Ala substitution introduced into pBAD33-$PAAR^{VSVG}$	This study
pBAD33 $PAAR_{C76A}{}^{-VSVG}$	Cys76-to-Ala substitution introduced into pBAD33-$PAAR^{VSVG}$	This study
pBAD33 $PAAR_{H14AC76}{}^{-VSVG}$	His14-to-Ala and Cys76-to-Ala substitution introduced into pBAD33-$PAAR^{VSVG}$	This study
pBAD33-$VgrG^{VSVG}$	T6SS-1 *vgrG* fused to a C-terminal VSVG tag cloned into pBAD33	(7)
pBAD33-VgrG1-386^{VSVG}	T6SS-1 *vgrG* (1-386) fused to a C-terminal VSVG tag cloned into pBAD33	(7)
pOK-$VgrG_{FLAG}$	T6SS-1 *vgrG* cloned into pOK12, C-terminal FLAG epitope	(2)
pACYC-$PAAR^{S}$	T6SS-1 *PAAR* cloned into pACYCDuet-1, C-terminal StrepTag II	This study
pBAD18-$PAAR^{VSVG}$	T6SS-1 *PAAR* cloned into pBAD18, C-terminal VSVG epitope	This study
pKOBEG	phage λ gam, *bet* and *exo* genes under the P*ara* promoter, Rep_{ts}, CmR	(8)
pKD4	KanR cassette flanked by FRT recombination sites, R6K origin, KanR, AmpR	(9)

Oligonucleotides

For mutant construction [a]

17-2 Δ*PAARΩKan* *(also called ΔPAARΩ)*

DEL-4537-5/DW TCCCTACGGCAACTGGTGACTCAAAGTGAAAATTTATTTTTGAGAGTATTTGTGTAGGCTGGAGCTGCTTCG

DEL-4537-3/DW TTATGAGGTAATTCTGTTGATAATGATTTTGTTCTGCTCCATCCCATGTTCATATGAATATCCTCCTTAGTTC

For plasmid construction [b, c, d]

pETDuet-^{S}VgrG 1-778 (pETDuet-^{S}VgrGΔTTR)

5-BamHI-sVgrG

ATT**GGATCC**AAGGAGATATACATATG*TGGAGCCACCCGCAGTTCGAAAAA*AATCTCACTGACTCCCTGCAAAATGTTTTATCCGG

3-HindIII-VgrGΔTTR GTAT**AAGCTT**TTACCCAAACCCTCGCGGCATTTC

pACYCDuet-PAAR^{FLAG}

5-NdeI-PAAR GATC**CATATG**AAGGAGATATACATATGTCTAAAGGTTTTGTATTGCTTGGTG

3-XhoI-PAARFL GTCG**CTCGAG**TTA*CTTGTCATCGTCGTCCTTATAATC*TCCTATTGCACATTCCGGTGCAC

pETDuet-$^{H\text{-}SUMO\text{-}}$PAAR

5- Bmt1-PAAR GATC**GCTAGC**TCTAAAGGTTTTGTATTGCTTGGT

3- HindIII-PAAR GTCG**AAGCTT**TTATCCTATTGCACATTCCGGTGC

pACYC-PAAR^{S}

5-NdeI-PAAR GATC**CATATG**TCTAAAGGTTTTGTATTGCTTGGTG

3-XhoI-PAARStrep GTCG**CTCGAG**TTA*TTTTTCGAACTGCGGGTGGCTCCA*TCCTATTGCACATTCCGGTGC

pBAD33-PAAR^{VSVG}

5pBAD-453**7** CTCTCTACTGTTTCTCCATACCCGTTTTTTTGGGCTAGCAGGAGGTATTACACCATGTCTAAAGGTTTTGTATTGCTTGGTG

3pBAD-4537-VSVG GGTCGACTCTAGAGGATCCCCGGGTACCTTA*TTTTCCTAATCTATTCATTTCAATATCTGTATA*TCCTATTGCACATTCCGGTG

For site-directed mutagenesis [e]	
**pACYC/pBAD33/pET^{H}SUMO PAAR$_{C41A}$**	
5’PAARCys41Ala	TTGTTGGAGATATGGTTAGTgcaCCTGTTTCCGGACATGGTAC
3’PAARCys41Ala	GTACCATGTCCGGAAACAGGtgcACTAACCATATCTCCAACAA
**pACYC/pBAD33/pET^{H}SUMO PAAR$_{C74A}$**	
5’PAARCys74Ala	TTGATGGATGTAAATGTCTGgcaGGGTGTAAGGTTATATCCAG
3’PAARCys74Ala	CTGGATATAACCTTACACCCtgcCAGACATTTACATCCATCAA
**pACYC/pBAD33/pET^{H}SUMO PAAR$_{C41AC74A}$**	
5’PAARCys41AlaCys74Ala	TTGTGATTGATGGATGTAAAgcaCTGgcaGGGTGTAAGGTTAT
3’PAARCys41AlaCys74Ala	ATAACCTTACACCCtgcCAGtgcTTTACATCCATCAATCACAA
**pACYC/pBAD33/pET^{H}SUMO PAAR$_{H46A}$**	
5’PAARHis46Ala	TTAGTTGCCCTGTTTCCGGAgcaGGTACCAATCCTATTGTGGA
3’PAARHis46Ala	TCCACAATAGGATTGGTACCtgcTCCGGAAACAGGGCAACTAA
**pACYC/pBAD33/pET^{H}SUMO PAAR$_{H14A}$**	
5’PAARHis14Ala	TGCTTGGTGATAATACCACAgcaGGTGGTAAAGTTATTTCTGC
3’PAARHis14Ala	GCAGAAATAACTTTACCACCtgcTGTGGTATTATCACCAAGCA
**pACYC/pBAD33/pET^{H}SUMO PAAR$_{C70A}$**	
5’PAARCys70Ala	GAGCTGTTGTGATTGATGGAgcaAAATGTCTGTGTGGGTGTAA
3’PAARHis70Ala	TTACACCCACACAGACATTTtgcTCCATCAATCACAACAGCTC
**pACYC/pBAD33/pET^{H}SUMO PAAR$_{C72A}$**	
5’PAARCys72Ala	TTGTGATTGATGGATGTAAAgcaCTGTGTGGGTGTAAGGTTAT
3’PAAR Cys72Ala	ATAACCTTACACCCACACAGtgcTTTACATCCATCAATCACAA
**pACYC/pBAD33/pET^{H}SUMO PAAR$_{C76A}$**	
5’PAARCys76Ala	GATGTAAATGTCTGTGTGGGgcaAAGGTTATATCCAGTGCACC
3’PAARCys76Ala	GGTGCACTGGATATAACCTTtgcCCCACACAGACATTTACATC

**pBAD33-PAAR (insertion of a STOP codon upstream VSVG coding sequence in pBAD33-PAAR^{VSVG})**	
FWSTOPBAD37-VSVG	CACCGGAATGTGCAATAGGATAaTACAGATATTGAAATGAATAG
RV STOPBAD37-VSVG	CTATTCATTTCAATATCTGTAtTATCCTATTGCACATTCCGGTG

[a] sequences corresponding to the downstream and upstream regions of the gene to be deleted underlined.
[b] sequence annealing to the target vector underlined.
[c] StrepTag II, 6His, FLAG or VSVG tag coding sequence italicized.
[d] restriction site in bold.
[e] mutagenized bases in lower case

Table S1 references

1. Zoued A, Durand E, Bebeacua C, Brunet YR, Douzi B, Cambillau C, Cascales E, Journet L. 2013. TssK is a trimeric cytoplasmic protein interacting with components of both phage-like and membrane anchoring complexes of the Type VI secretion system. *J Biol Chem* 288: 27031-41.

2. Flaugnatti N, Le TT, Canaan S, Aschtgen MS, Nguyen VS, Blangy S, Kellenberger C, Roussel A, Cambillau C, Cascales E & Journet L. 2016. A phospholipase A1 antibacterial Type VI secretion effector interacts directly with the C-terminal domain of the VgrG spike protein for delivery. *Mol Microbiol* 99: 1099-1118.

3. Brunet YR, Zoued A, Boyer F, Douzi B, Cascales E. 2015. The Type VI Secretion TssEFGK-VgrG Phage-Like Baseplate Is Recruited to the TssJLM Membrane Complex via Multiple Contacts and Serves As Assembly Platform for Tail Tube/Sheath Polymerization. *PLoS Gene*t 11:e1005545.

4. Durand, E., Nguyen, V., Zoued, A. *et al.* 2015. Biogenesis and structure of a type VI secretion membrane core complex. *Nature* 523:555–560.

5. Guzman LM, Belin D, Carson MJ, Beckwith J. 1995. Tight regulation, modulation, and high-level expression by vectors containing the arabinose PBAD promoter. *J Bacteriol* 177: 4121-30.

6. Flaugnatti N, Rapisarda C, Rey M, Beauvois SG, Nguyen VA, Canaan S, Durand E, Chamot-Rooke J, Cascales E, Fronzes R, Journet L. 2020. Structural basis for loading and inhibition of a bacterial T6SS phospholipase effector by the VgrG spike. *EMBO J* 39: e104129.

7. Renault M, Zamarreño Beas J, Douzi B, Chabalier M, Zoued A, et al. 2018. The gp27-like Hub of VgrG Serves as Adaptor to Promote Hcp Tube Assembly. *J Mol Biol* 430:3143-3156.

8. Chaveroche MK, Ghigo JM, d'Enfert C. 2000. A rapid method for efficient gene replacement in the filamentous fungus Aspergillus nidulans. *Nucleic Acids Res 28*, e97.
9. Datsenko KA, Wanner BL. 2000. One-step inactivation of chromosomal genes in Escherichia coli K-12 using PCR products. *Proc Natl Acad Sci U S.A*. 97: 6640–6645
